# Expression of FACT in mammalian tissues suggests its role in maintaining of undifferentiated state of cells

**DOI:** 10.18632/oncotarget.340

**Published:** 2011-10-13

**Authors:** Henry Garcia, Daria Fleyshman, Katerina Kolesnikova, Alfiya Safina, Mairead Commane, Geraldine Paszkiewicz, Angela Omelian, Carl Morrison, Kateryna Gurova

**Affiliations:** ^1^Department of Cell Stress Biology, Roswell Park Cancer Institute, Elm and Carlton Streets, Buffalo, NY, 14263; ^2^Department of Pathology, Roswell Park Cancer Institute, Elm and Carlton Streets, Buffalo, NY, 14263

**Keywords:** chromatin remodeling, transcription, SPT16, SSRP1, cancer, differentiation, proliferation, oncotarget

## Abstract

The Facilitates Chromatin Transcription (FACT) chromatin remodeling complex, comprised of two subunits, SSRP1 and SPT16, is involved in transcription, replication and DNA repair. We recently showed that curaxins, small molecules with anti-cancer activity, target FACT and kill tumor cells in a FACT-dependent manner. We also found that FACT is overexpressed in human and mouse tumors and that tumor cells are sensitive to FACT downregulation. To clarify the clinical potential of FACT inhibition, we were interested in physiological role(s) of FACT in multicellular organisms. We analyzed SSRP1 and SPT16 expression in different cells, tissues and conditions using Immunohistochemical (IHC) staining of mouse and human tissues and analysis of publically available high-content gene expression datasets. Both approaches demonstrated coordinated expression of the two FACT subunits, which was primarily associated with the stage of cellular differentiation. Most cells of adult tissues do not have detectable protein level of FACT. High FACT expression was associated with stem or less-differentiated cells, while low FACT levels were seen in more differentiated cells. Experimental manipulation of cell differentiation and proliferation in vitro, as well as tissue staining for the Ki67 proliferation marker, showed that FACT expression is related more to differentiation than to proliferation. Thus, FACT may be part of a stem cell-like gene expression signature and play a role in maintaining cells in an undifferentiated state, which is consistent with its potential role as an anti-cancer target.

## INTRODUCTION

FACT (Facilitates Chromatin Transcription) is a chromatin remodeling complex composed of two subunits, Structure Specific Recognition Protein 1 (SSRP1) and Suppressor of Ty 16 (SPT16). We have identified FACT as a molecular target of a novel class of candidate anti-cancer agents named curaxins [1]. Curaxin-induced “trapping” of FACT within chromatin alters FACT's functions in tumor cells, resulting in activation of the pro-apoptotic p53 pathway, suppression of the anti-apoptotic NF-κB pathway, and FACT-dependent tumor cell death [1]. We also found that expression of both FACT subunits was elevated in several types of mouse and human tumor cell lines as compared to their normal counterparts and that genetic knockdown of either FACT subunit compromised tumor cell viability [1]. These data suggest that FACT might play a role in development, maintenance or progression of cancer and, therefore, be a potential target for anti-cancer therapy via curaxins or other agents. However, an improved understanding of the physiological role(s) of FACT under normal conditions as well as its pattern of expression in mammals is necessary before the consequences of global FACT inhibition can be predicted and development of anti-FACT therapeutic approaches can proceed.

There are indications in the literature that FACT may be expressed at constantly high levels in a “housekeeping” fashion, similar to basic transcription factors. For example, yeast growth was prevented by inactivating mutations in either FACT subunit [2-4], knockdown of the SSRP1 subunit of FACT in mice caused death on day E3.5 at the blastocyst stage [5], and SSRP1 was also shown to be essential for *Arabidopsis* viability [6]. In addition, the model systems used to study the biochemical function of FACT in mammals (primarily HeLa cells) and yeast typically have very high levels of FACT expression. However, a limited number of other studies have shown that expression of at least SSRP1 is not ubiquitous among tissues of higher eukaryotes. First, it was shown that only highly proliferative mouse tissues express detectable SSRP1 RNA and protein and that SSRP1 levels decline upon induction of differentiation in vitro [7]. Second, indirect immunofluorescence analyses revealed co-localization of both FACT subunits in nuclei of the majority of cell types in *Arabidopsis thaliana* embryos, shoots, and roots, while FACT was not present in terminally differentiated cells such as mature trichoblasts and cells of the root cap [8].

Although FACT is involved in transcription not all types of transcription depend on FACT. In human tumor cells knockdown of both FACT subunits changed expression of less than 200 genes more than 2 times [9]. In yeast FACT assisted transcription of genes with highly ordered chromatin structure and induced genes, but not constantly expressed housekeeping genes [10]. This suggests that FACT may not belong to the category of general transcriptional factors and may be required for only certain subtypes of transcription. Identification of a set of genes which requires FACT for transcription is hampered by the fact that cells in vitro are not viable upon knockdown of FACT [1]. Therefore as a first step to approach FACT dependent transcriptional program we seek to identify conditions which may require high level of FACT expression in cells.

**Table 1:** Expression of FACT subunits in different organs of mouse and human

Organ/system	cells	mouse	human	Organ/system	cells	mouse	human
**hematological**	lymphocytes	++	++	**large intestine**	bottom crypt cells	+++	+++
	macrophages/monocytes	+++	+++		surface epithelial cells	-	-
	granulocytes	-	NA		stroma	-	-
	reticulocytes	-	NA	**lung**	alveolar epithelia	-	-
	spleen	++	++		air ducts epithelia	+/-	-
	bone marrow	+++	NA		stroma	-	-
	MALT	++	NA	**mammary gland**	epithelia	+	-
	thymus	+++	NA		adipose cells	+	-
**liver**	hepatocytes	-	-	**nervous system**	grey matter neurons	+/-	+/-
	stroma	-	-		white matter	+	+
**kidney**	nephrons	-	-		glia	-	-
	proximal tubular epithelia	-/+	-/+		Purkinje cells	++	NA
	distal tubular epithelia	-/+	-		granule cells	-	
	stroma	-	-		peripheral neurons	-	NA
**pancreas**	acinar cells	-	-	**different organs**	endothelial cells	-	-
	ductal cells	+/-	-		fibroblasts	-	-
	Langerhans islets	++	-		muscles	-	-
	stroma	-	-		adipocytes	-	-
				**ovary**	*See details in the text*	+++	+++
**stomach**	bottom crypt cells	+++	+++	**testes**	*See details in the� text*	+++	+++
	surface epithelial cells	-	-	**endometrium**	*See details in the text*	+++	+++
	stroma	-	-	**prostate**	basal cells	+/-	+/-
**small intestine**	bottom crypt cells	+++	+++		luminal cells	-	-
	villi epithelial cells	-	-		stroma	-	-
	stroma	-	-				

We did this through the analysis of FACT subunits expression in different mammalian (mouse and human) tissues and cells under different conditions to better understand the physiological role(s) of FACT and the potential implications of its targeting by anticancer therapeutics. Our approach was based on the presumption that conditions associated with high FACT levels would be more likely to be dependent on FACT function than conditions with low or absent FACT expression. The same assumptions can be applied to cell types and tissues differing in FACT expression levels.

Two methods were used to map FACT subunit expression in mammals. First, immunohistochemical (IHC) staining of normal human and mouse tissue sections was performed using antibodies against SSRP1 (human and mouse) and SPT16 (mouse only). This analysis demonstrated that FACT subunits are not ubiquitously expressed. On the contrary, FACT was expressed at very low or undetectable levels in most adult tissues with a few exceptions. The second method took advantage of the wealth of mRNA expression data available in the NCBI Gene Expression Omnibus (GEO) database. We analyzed all available datasets in which either SSRP1 or SPT16 was measured in mammalian cells or tissues. This demonstrated that high or low FACT subunit expression is not stochastically distributed among different experiments, but is associated with certain conditions. Western blotting and immunofluorescence were used to confirm the findings of this data mining. Overall, the two strategies for FACT expression analysis were concordant and demonstrated association of “high” FACT expression with the condition of “stemness” or undifferentiated states such as embryonic stem cells, germ cells, and progenitors of different types of cells. Accordingly, FACT levels were lower in more differentiated states. All of the observed “high-FACT” cell states/conditions are characterized by the ability of cells to renew themselves. However, we used staining of Ki67 proliferation marker as well as in vitro systems for experimental modulation of proliferation and differentiation to show that FACT expression is not directly associated with the proliferative status of cells or tissues.

## RESULTS

### Expression of FACT subunits in normal mammalian tissues is not ubiquitous

Expression of the SSRP1 and SPT16 subunits of FACT was studied by immunohistochemistry in normal tissues of adult mice (FVB, 8-12 weeks old, male and female) and humans (control non-cancerous tissues from cancer patient on tumor tissue microarrays (TMA) provided by RPCI Pathology Network Recourse). A summary of the results is presented in Table 1. In general, the pattern of SSRP1 and SPT16 expression was similar between mouse and human tissues of both genders. We also observed strong similarity in the distribution of SSRP1 and SPT16 staining on most of the slides. This suggests that the two FACT subunits are expressed in a coordinated manner, which is consistent with their known function as a heterodimeric complex and with a previously reported study in plants [8].

**Figure 1:**
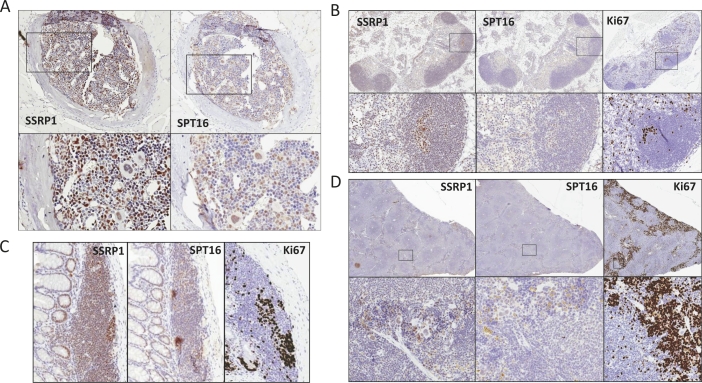
Expression of FACT subunits in hematological and lymphoid organs Immunohistochemical staining with antibodies against SSRP1, SPT16 and Ki67 (except bone marrow) of A. - bone marrow; B. –lymph node, C.- MALT; D. – spleen. Areas in squares are enlarged below each image.

The majority of adult human and mouse tissues did not stain positively for either FACT subunit (Table 1 and Fig.S1). However, high levels of SSRP1 and SPT16 were observed in some cells of the bone marrow (Fig. 1A), and thymus; in lymph nodes (with the highest levels observed in germ centers of lymphoid follicles; Fig. 1B), and among lymphocytes of mucosa-associated lymphoid tissue (MALT; Fig. 1C). Both SSRP1/SPT16-positive and -negative cells were detected in the spleen with more or less even distribution between the red and white pulp (Fig. 1D).

**Figure 2:**
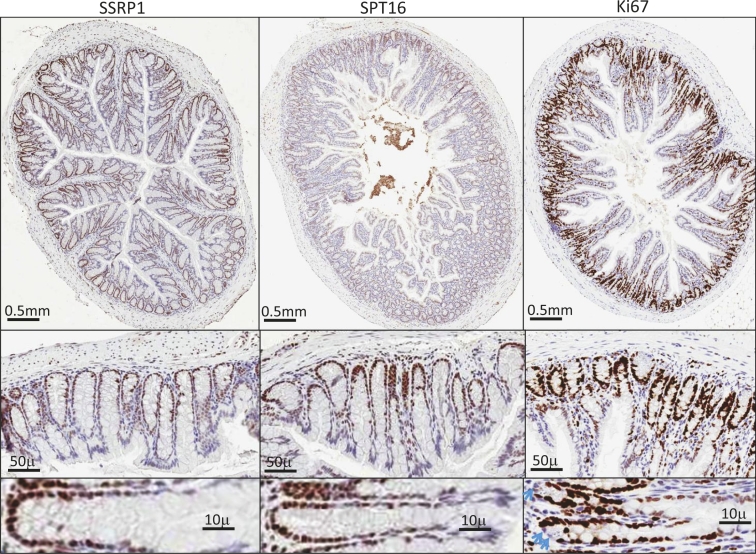
Expression of FACT and proliferation marker Ki67 in intestine Immunohistochemical staining of a section of small intestine with antibodies against SSRP1, SPT16 and Ki67. Blue arrows show Ki67 negative cells at the bottom of a crypt.

**Figure 3:**
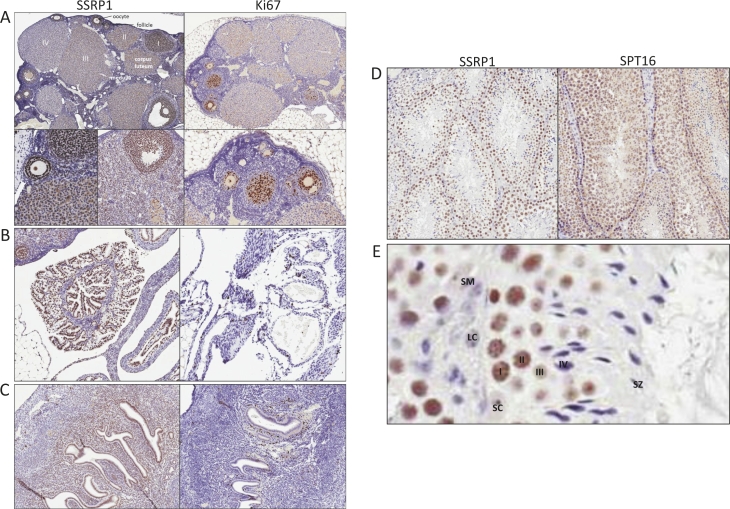
Expression of FACT in reproductive organs A-C. Immunohistochemical staining of a section of ovary (A), infundibulum of fallopian tube (B), uterus (C) with antibodies against SSRP1and Ki67. D. Immunohistochemical staining of a section of testes with antibodies against SSRP1and SPT16. Note the orderly maturation of germ cells from the base to the center of the lumen. E. Higher magnification of a portion of lumen in testes stained with anti-SSRP1 antibody. Spermatogonia (I, along the basement membrane), primary (II) & secondary (III) spermatocytes, spermatids, (IV) and spermatozoa (SZ) are shown. SM – smooth muscle cells, LC – Leydig cells, SC – Sertoli cells.

**Figure 4:**
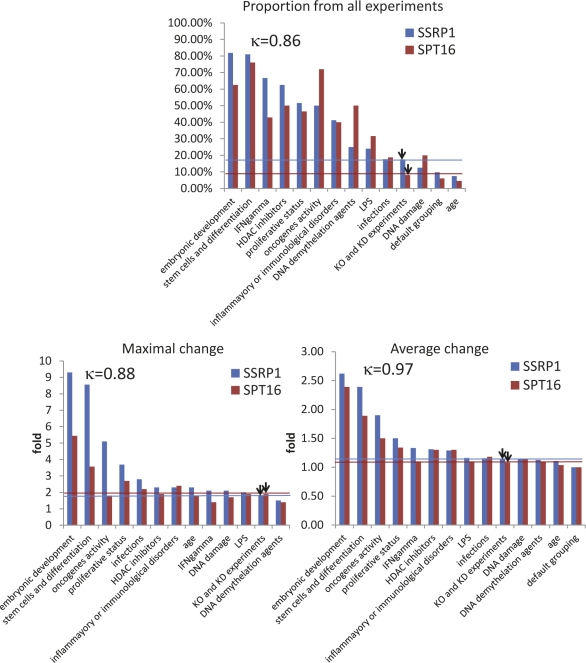
Identification of conditions associated with changes in FACT subunit expression through analysis of the GEO database Experimental conditions associated with either SSRP1 or SPT16 expression level changes were selected and ranked based on (i) the proportion of all experiments testing similar conditions in which the level of mRNA for either FACT subunit was changed (proportion of all experiments), (ii) the maximal level of change (maximal change), and (iii) the average level of change (average change). Cut-off lines for SSRP1 and SPT16 in corresponding colors were drawn using parameters generated for experiments where knockout or knockdown tissues or cells were used (technical classification). In the left upper corner of each plot, the Pearson correlation coefficient between changes in SSRP1 and SPT16 expression levels is shown.

One of the cell types showing the strongest FACT subunit staining were the epithelial cells at the bottom of crypts in all parts of the intestine and in the stomach, with staining gradually decreasing up to the top of crypt (Fig.2). Epithelial cells of villi and stromal cells of the intestine were negative for FACT expression as were stromal cells of all other organs tested (Fig. 1-4, S1 and Table 1).

Many SSRP1/SPT16-positive cells were observed in ovary and uterus. However, we were not able to reliably interpret the data for SPT16 due to strong cytoplasmic background staining. Therefore, we only describe SSRP1 distribution in these organs. The cells of the mouse ovary (no full size human ovary were stained) showing the strongest SSRP1 staining were follicle cells. Oocytes were also positive. It appeared that transformation of a follicle into a corpus luteum was accompanied by a decline in SSRP1 expression. Within an ovary, there was a gradient of SSRP1 staining between different corpi with levels ranging from equal to that seen in follicle cells to nearly SSRP1-negative (Fig. 3A, compare corpi I, II, III and IV). Ovarian medulla cells were less SSRP1-positive and in some cases were nearly SSRP1-negative. Stromal cells were negative. The distribution of SSRP1-positive cells with ovarian tissue suggests that that there is a cycle of SSRP1 expression in the mouse ovary, probably coinciding with the developmental cycle of ovarian cells.

Endometrial cells of the uterus and epithelial cells of the fallopian tubes were found to be strongly SSRP1-positive, with the most intense staining observed among fimbriae cells of the infundibulum (Fig.3B, C). Both SSRP1-positive and -negative cells were seen in the myometrium (Fig.4C).

Differential expression of SSRP1/SPT16 was also observed within testes, with spermatogonia cells being the most positive, spermatocytes showing less intense staining, and supporting cells (Leydig and Sertoli cells) and stroma being negative (Fig. 4A). As in the ovary, this pattern is suggestive of FACT expression changing in association with the developmental cycle of male reproductive cells.

Most of the cells in the liver and pancreas were SSRP1/SPT16-negative, although there were some weakly positive cells among Langerhans islets (Fig. S1a,d and c,f). Among other endocrine tissues, the cells of the zona glomerulosa in the adrenal cortex were found to express SSRP1 (Fig.S1i,l). These cells produce mineralcorticoids. Other cells of the adrenal cortex, as well as cells of the adrenal medulla, did not show detectable SSRP1 or SPT16 staining. Lung epithelial and stromal cells were negative (Fig.S1 b,e). Some kidney tubular epithelial cells were moderately positive, with the frequency of positivity increasing in more distal regions (Fig.S1g,j). SSRP1/SPT16-positive cells were also observed among brain neurons, such as Purkinje cells of the cerebellum (Fig. S1h,k), although the CNS was not analyzed in detail.

Taken together these data indicate that FACT subunits are not ubiquitously expressed at high levels in any mammalian tissue. Even in those tissues showing the highest frequency and intensity of FACT staining (primarily lymphoid and reproductive organs), a significant number of negative cells were also observed.

### Expression of FACT subunits and the Ki67 proliferation marker are not correlated

The mosaic distribution of FACT expression in lymphoid and some other organs suggested that it might be related to the proliferation status of cells. Although this hypothesis clearly could not be true for all tissues (e.g., FACT-positive Purkinje cells in cerebellum do not proliferate), we tested it by comparing serial mouse tissue sections stained for Ki67 antigen (a well-established marker of proliferation) and FACT subunits.

There was no substantial similarity between FACT and Ki67 expression in any of the hematological and lymphoid organs assessed. For example, nearly all cells in lymph node follicles and MALT were FACT-positive, but only a limited number were Ki67-positive (Fig. 1B, C). Moreover, the strongest FACT staining in these structures did not coincide with Ki67 positivity, although co-staining of the same slide is needed to make a final conclusion. In the spleen, there was not a substantial difference in FACT expression between the red and white pulp; however, the frequency of strongly Ki67 positive cells was much greater in the red pulp than in the white pulp (Fig. 1D).

The highest coincidence of SSRP1 and Ki67 positivity was seen in the intestinal epithelium (Fig.2). Intestinal stem cells and highly proliferative progenitor cells reside at the bottom of intestinal crypts and the proliferation potential of the cells decreases as they move towards the top of a crypt. This was reflected by the pattern of Ki67 staining. Importantly, the cells at the very bottom of a crypt are actual intestinal stem cells, which proliferate more slowly than epithelial progenitors located 2-3 cells higher up in the crypt (Fig.3). This explains the observation of one or two Ki67-negative cells at the bottom of many crypts. However, no similar FACT-negative cells were seen at the bottom of most crypts, again suggesting that although the pattern of FACT and Ki67 staining in all parts of the intestine is similar, it is not identical.

The ovary and uterus also showed similarity, but not identity, in distribution of FACT- and Ki67-positive cells. We observed a gradient of FACT positivity in ovaries with the highest expression seen in follicles and oocytes and the lowest expression seen in old corpi lutei and medulla cells. In contrast, only cells of the inner mass of follicles were Ki67 positive. In the uterus, Ki67-positive cells were detected only in the endometrium, while many FACT-positive cells were present in the myometrium as well as the endometrium (Fig.3).

Taken together, these data indicate that expression of FACT subunits is not directly related to the proliferative status of cells in the organs analyzed.

### SSRP1/SPT16 mRNAs and proteins are distributed in similar patterns in normal tissues

There are several microarray studies where SSRP1/SPT16 mRNA expression was compared between different normal human and mouse tissues (Table S1). First what we noticed that mRNA of both subunits in contrast to proteins were easily detectable and expressed at quite substantial level in almost all adult tissues tested. There was less variability in expression of SSRP1/SPT16 mRNAs between different adult tissues than we observed for SSRP1/SPT16 proteins (maximal difference for mRNAs was < 3-fold). As in our IHC analyses, we observed that expression levels of SSRP1 and SPT16 mRNAs were generally concordant, although there were some discrepancies (e.g., study GDS565 showed high expression of SPT16, but not SSRP1, in the hypothalamus, Table S1).

**Table 2:** Classification of studies which demonstrated differential level of FACT subunits between experimental conditions

Category	Keywords	Studies measuring SSRP1 level					Studies measuring SPT16 level				
		number of similar studies (datasets)	number of studies showing change in SSRP1 level	%	maximal change	average	number of similar studies (datasets)	number of studies showing change in SPT16 level	%	maximal change	average
**embryonic development**	embryo, prenatal	11	9	**81.82%**	9.3	2.62	9	6	**66.67%**	5.44	2.39
**IFNgamma**	IFNgamma	9	6	66.67%	2.1	1.33	7	3	**42.86%**	1.4	1.1
**HDAC inhibitors**	HDAC	8	5	62.50%	2.3	1.31	6	3	**50.00%**	1.9	1.3
**stem cells and differentiation**	stem, precursor, progenitor, differentiation	63	51	80.95%	8.56	2.39	46	35	**76.09%**	3.57	1.89
**proliferative activity**	proferation, division, growth	31	16	51.61%	3.69	1.50	28	13	**46.43%**	2.69	1.34
**inflammatory or immunological disorders diseases**	manual search	17	7	**41.18%**	2.3	1.29	15	6	**40.00%**	2.4	1.3
**oncogenes activity**	manual search	14	7	50.00%	5.1	1.85	11	8	**72.73%**	4.4	1.9
**DNA demythelation agents**	demythelation	4	1	25.00%	1.5	1.13	2	1	**50.00%**	1.4	1.1
**LPS**	LPS	25	6	24.00%	2	1.16	19	6	**31.58%**	1.9	1.1
**infections**	infection	51	9	17.65%	2.8	1.16	32	6	**18.75%**	2.2	1.18
**DNA damage**	manual search	8	1	12.50%	2.1	1.14	5	1	**20.00%**	1.7	1.14
**age**	age	27	2	**7.41%**	2.3	1.11	22	1	**4.55%**	1.8	1.036364
**all experiments**	NA	3686	387	**10.50%**	NA	1	2418	6	**0.25%**		1
**KO and KD experiments**	NA	131	23	17.56%	1.82	1.15	99	8	**8.08%**	1.9	1.08

Organs in which expression of SSRP1 was higher than in other tissues (2 times higher than mean of all tissues in a study) in most of the analyzed studies (both human and mouse) were testis and ovary, followed by thymus and uterus. In mouse, higher than average expression of SSRP1 was also observed in spleen and mammary gland. Tissues in which high expression of SSRP1 mRNA was observed in at least one study were bone marrow and trachea (both species), placenta, bladder, and tonsils (human only), and lymph node, bone, and umbilical cord (mouse only).

Like SSRP1, SPT16 mRNA was most frequently detected at higher than average levels in testis and ovary. In addition, single studies showed elevated SPT16 expression in human thymus, mammary gland, skeletal muscle, heart and peripheral blood lymphocytes and in mouse spleen and hypothalamus.

Overall, the data from these previously reported studies show that, for both mouse and human, SSRP1 and SPT16 mRNA expression is highest in tissues of hematological and reproductive systems. This is consistent with the patterns of SSRP1 and SPT16 protein abundance that we observed via IHC staining (see above).

### Identification of conditions associated with changes in FACT expression

Existing mRNA expression data was not only useful for examining the tissue distribution of FACT expression (see above), but also allowed us to identify specific conditions associated with altered FACT expression. We accomplished this by analyzing mRNA expression data for both FACT subunits from a large number of independent studies available at NCBI Gene Expression Omnibus (GEO) site [11, 12]. To facilitate data interpretation, we limited our analysis to mammalian species. We found a total of 3686 entries for SSRP1 and 2418 entries for SPT16 (roughly 2000 experiments in which expression of at least one subunit was measured). The number of entries for SSRP1/SPT16 in different species was as follows: 1462/1106 – *H. sapiens*, 1746/1140 – *M. musculus*, 269/191 – *R. norvegicus*, 13/6 – *M. mulata*, 16/16 – *C. lupus*, 2/2 – *B. taurus*. This approach was expected to provide unbiased detection of physiological roles of FACT since the analyzed studies were generally global gene expression profiling studies that tested a wide array of different conditions without specific focus on FACT.

Although there are several software programs available for analysis of multiple sets of microarray data, most are designed to compare healthy versus diseased conditions (e.g., Oncomine) and those that are capable of analyzing changes in expression between other conditions were not satisfactory due to the limited accuracy of the classification of conditions that they provide. Therefore, we performed a “manual” analysis of the GEO database using the GEO Profiles search engine (see Materials and Methods for details). Briefly, we first selected all experiments in which expression of either SSRP1 or SPT16 was different between any conditions (for criteria see Material and Methods). We then classified all conditions in which expression of SSRP1/SPT16 was changed according to the biological process involved (see Table 2), such as embryonic development, differentiation, treatment with a certain compound, etc. We then performed another GEO Profiles search using combinations of SSRP1/SPT16 (or Supt16h, official name of gene) and keywords describing biological processes used for classification (embryo, stem cell, differentiation, compound name etc). This process revealed the proportion of experiments in which expression of SSRP1/SPT16 was changed in similar conditions from all experiments testing the same conditions. For example, out of a total of 25 experiments in which SSRP1 gene expression was compared between LPS-stimulated and control cells, a change in SSRP1 expression level after LPS stimulation was observed in 6 experiments and no change was observed in 19 experiments.

Through this process, we identified (i) conditions in which FACT levels were changed most frequently (based on the proportion of experiments showing a change in FACT expression out of all experiments with similar conditions); (ii) conditions associated with the maximal change in FACT levels; and (iii) the average change in FACT levels in experiments with similar conditions. All experimental conditions associated with altered FACT expression were ranked according to these parameters. To establish a cut-off line to distinguish conditions in which FACT levels were changed with higher than background frequency, we used two approaches: (i) we calculated the same parameters (except maximal change) for all experiments that measured FACT levels; and (ii) we calculated the same three parameters for a list of mRNA expression experiments classified according to technical rather than biological principles (i.e., experiments with knockout or knockdown of any gene). We used the highest number generated by either approach as the cut-off for each parameter.

**Figure 5:**
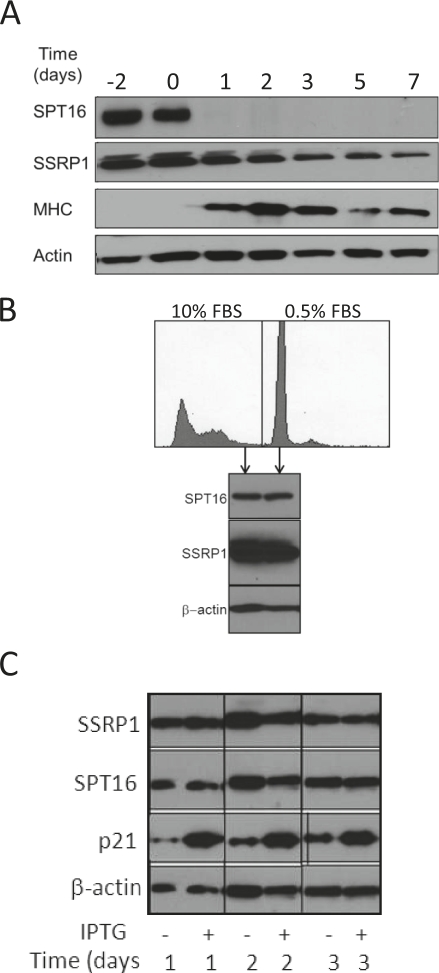
FACT level is changed upon differentiation and transformation, but not with a change in the proliferation status of cells A. C2C12 myoblast cells were grown to confluency in regular growth medium. At time 0, when cells were confluent, the medium was changed to differentiation medium. Cell samples were collected on the indicated days before and after induction of differentiation and the level of SSRP1, SPT16, myosin heavy chain (MHC1, differentiation marker) were assessed using western blotting. β-actin was assessed as a loading control. B. HT1080 fibrosarcoma cells were grown to confluency and then placed into low serum medium (0.5% FBS) to induce G1 arrest. Forty-eight hours later, SSRP1 and SPT16 proteins were detected by Western blotting and DNA content was measured by FACS analysis of fixed propidium iodide- stained cells. C. Effect of induced expression of CDK inhibitor p21/Waf1 on the level of FACT subunits. Western blotting of HT1080 cells with IPTG-regulated expression of p21/Waf1 collected at different time points after addition of IPTG.

Based on all three parameters, two conditions, “embryonic development” and “stem cells and differentiation”, were most clearly associated with changes in FACT expression level (Fig.4). We identified other conditions in which all three parameters were higher than the cut-off (e.g., “proliferative activity“ and “expression of oncogenes”), but they were much more weakly associated with FACT expression than the first two conditions (Fig.5). There were high positive correlation between changes in SSRP1 and SPT16 level for all conditions (κ>0.85).

### FACT is expressed at different levels in differentiated and non-differentiated cells

Having identified conditions associated with changes in FACT expression, the next step was to determine whether the change in FACT level in a given condition was in the same or opposite direction (increase or decrease) in different experiments (the initial selection did not distinguish the direction of the change). Our findings for the categories of “embryonic development”, “stem cells and differentiation”, “proliferative activity“, and “expression of oncogenes” are described below.

Table 3 summarizes all of the experiments in which FACT levels were measured at different stages of embryonic development. In all cases except for one, when SSRP1 levels showed a change, it was lower at later stages of embryonic or post-embryonic development than at earlier stages (Fig.S2B-C). The single case in which this was not observed was in pre-implantation embryos. In this case, SSRP1 expression was low in zygote and elevated towards the blastocyst (Fig.S2A). Changes of SPT16 were similar except that expression of SPT16 was not increased in the blastocyst as compared with earlier stages of pre-implantation embryos (Table 3). Therefore, this analysis of data from multiple microarray experiments using different tissues indicates that FACT expression increases during development of the pre-implantation embryo up to at least the blastocyst stage and then declines during the course of embryonic development.

**Table 3:** Summary of studies measured levels of FACT subunits in embryonic tissues

GEO Dataset Study	Tissue Compared	Species	SSRP1			SPT16 (=Supt16h)		
			description of change	fold change	p-value	description of change	fold change	p-value
**GDS2577**	liver	M. musculus	higher in embryo versus adult	9.30	3.6233E-39	higher in embryo versus adult	5.44	1.8437E-26
**GDS1511**	whole embryos	M. musculus	higher at E8.5 than at E12	2.62	0.026863	higher at E8.5 than at E12	3.29	0.038166
**GDS813**	preimplantation embryos	M. musculus	highest in blastocyst	2.44	4.7105E-08	no change between oocyte and blastocyt	1.00	-
**GDS2283**	brain	M. musculus	higher in embryo at E13.5 than in newborn	2.16	0.000113	higher in embryo at E13.5 than in newborn(not statistically significant	1.37	0.059308
**GDS782**	lungs	M. musculus	higher at at E18.5 than at birth	2.14	0.02756044	No data		
**GDS827**	heart	M. musculus	decrease from E10.5 to E18.5	2.08	<0.000001	decrease from E.10.5 to E18.5	1.41	0.000046
**GDS3442**	brain	M. musculus	higher at E9.5 than at E13.5	1.69	<0.000001	higher at E9.5 than at E13.5	inconsistent data	-
**GDS3641**	yolk sac	M. musculus	higher at E9 than at E10.5	1.53	2.787E-05	higher at E9 than at E10.5	1.84	0.000056
**GDS1724**	gonadal somatic cells from male and female embryos	M. musculus	higher at E10.5 than at E11.5 in males	1.26	0.000001	No difference	-	-
**GDS739**	fetal orofacial tissue	M. musculus	no change between E12- E14 (unrealible data)	1	-	No data		
**GDS1003**	bovine early embryo	B. Taurus	no clear change big variability (one panel)	-	-	no clear change big variability (one panel)	-	-

Table S2 summarizes experiments in which FACT expression was compared in cells at different stages of differentiation. In 45 out of 51 (88%) SSRP1 studies and in 33 out of 35 (94%) SPT16 studies, expression of FACT subunits were lower in more differentiated cells than in stem cells, progenitor cells and less differentiated cells (Fig.S3). FACT expression declined after induction of differentiation (Fig. S3A, C-F) and increased after induction of regeneration (Fig.S3B). Very few studies showed increased expression of SSRP1 (6 out of 51, 12%) or SPT16 (2 out of 35, 6%) in differentiated cells as compared to progenitor cells (Table S2, entries highlighted in red).

It is difficult to completely separate in vitro conditions that induce differentiation from those that cause pure growth arrest. Therefore, we excluded from our “proliferation activity” group all studies where “differentiation” was mentioned (Table S3). Although this is not ideal approach it allowed formal separation of these two phenomena for the analysis. SSRP1/SPT16 expression was changed in 52%/46% of the remaining “proliferation-specific” experiments in which cells of different proliferative status were compared, respectively. The majority of studies (88%/69%) that showed a proliferation-associated change in SSRP1/SPT16 expression showed a decrease in SSRP1/SPT16 in conditions of growth arrest or an increase in SSRP1/SPT16 in conditions of active proliferation (Tables 2 and S3). From this analysis we concluded that although there association of high FACT and proliferation and low FACT and quiescence this association was seen only in less than half of studies (i.e. SSRP1 higher in proliferating cells than in arrested in 49% of studies (88 % of 56% of total studies measuring proliferation) and SPT16 in 32% (69% of 46% of total studies). Therefore, similar to our IHC experiments, this analysis allowed us to conclude that changes in proliferative status are not strictly associated with changes in FACT expression.

Finally, we analyzed how the activity of oncogenes influences FACT expression levels. In 71%/69% of experiments in which oncogene activity was induced in different cells in vitro or in vivo, SSRP1/SPT16 levels were increased (Table S4). In contrast to induction of proliferation, there were no examples of reduction of FACT level in cells with elevated oncogene activity. Among the oncogenes assessed in the available studies were N-myc, mutated RAS or activated MEK, Gli1 and Smoothed, SV40.

Other experimental conditions associated with less prominent changes in FACT expression included treatment with LPS, IFNγ, or HDAC inhibitors and DNA demethylation or damage (Table 2 and Fig. 4). Determination of the significance of these associations will require additional investigation.

### FACT subunit expression levels are changed upon experimental induction of differentiation, but not proliferation

The RNA expression and IHC data described above suggested that FACT expression levels are related to the differentiation status of cells. To directly test whether FACT expression changes during the process of differentiation, we evaluated FACT protein levels in an in vitro system in which cultures of C2C12 myoblasts are induced to differentiate into myotubes by growth in a special medium (“differentiation medium”). Reduction of FACT mRNA levels upon differentiation in this model was previously demonstrated in several microarray hybridization experiments (Fig. S3C) and another study showed reduction of SSRP1 protein level but did not assess SPT16 [7]. This model also had the potential to reveal whether FACT levels change as a result of decreased proliferation or induced differentiation, since before addition of differentiation medium, the cell cultures are grown to high density and the cells become growth arrested due to contact inhibition [13]. We did not observe any change in FACT protein levels (either subunit) during the period of culture growth before addition of differentiation medium (days -2-0). In contrast, after addition of differentiation medium on day 0, there was a clear gradual decrease in SSRP1 and a sharp reduction in SPT16 protein levels in parallel with increased expression of myosin heavy chain, a marker of C2C12 differentiation (Fig. 5A). These results provide additional support for our conclusion that FACT expression is related to differentiation status, not proliferation status.

We further tested whether FACT levels are associated with changes in the proliferative status of cells by using several independent methods to arrest the growth of human fibrosarcoma HT1080 cells (i.e., growth to high density/contact inhibition, incubation of cells in medium with low serum or ectopic expression of the CDK inhibitor, p21/Waf1). No changes in SSRP1 or SPT16 expression were observed under any of these conditions (Fig. 5B, C), again providing support for a lack of direct association between proliferation and FACT expression.

Overall, these experiments with cultured cells provided confirmation on the protein level of tendencies that we observed on the mRNA level in our analysis of publically available microarray data, and, therefore, validated our approach.

## DISCUSSION

Known roles of FACT include facilitating transcription from nucleosomal templates, DNA damage responses, and V-D-J recombination [3, 4, 14]. Many mechanistic details of FACT's involvement in these processes have been uncovered (for review see [15, 16]. For example, it is well-established that FACT plays a unique role in regulating assembly of nucleosomes and exchange of some types of H2 histones. These activities influence transcription and DNA damage responses. However, absence of FACT subunits does not have a general inhibitory effect on transcription, but only interferes with expression of a subset of genes [8-10]. It has been postulated that those transcriptional programs that are FACT-dependent are likely tightly regulated, non-housekeeping programs and that remodeling of chromatin by FACT provides one level of their control. For example, we showed that transcription by the stress-responsive transcription factor NF-κB requires FACT activity [1].

In large part, the cell type- and tissue-specificity of FACT function has not been previously explored. The functional consequences of FACT inhibition in different types of cells were not studied. Moreover, most FACT studies were run in yeast or human tumor cells in which FACT subunits are abundantly expressed. A limited number of studies showed that some differentiated cells express little or no FACT [7], [8]. These studies, as well as reports implicating FACT in replication [2, 17], recombination [18] and mitosis [19], suggest that FACT expression levels may be associated with the proliferative status of cells. However, we observed that although mouse and human diploid fibroblasts actively proliferate in vitro, they have very low or nearly undetectable levels of FACT [1].

Due to our discovery that candidate anti-cancer compounds Curaxins cause functional inactivation of FACT [1], we were interested in gaining a better understanding of the role(s) of FACT in the context of mammalian organisms. Since FACT function requires that it is expressed, we performed a detailed investigation of FACT expression in different organs and tissues of mice and humans as a first step toward identification of FACT's biological roles. We found that expression of the two subunits of FACT is highly correlated on both the RNA and protein levels, consistent with their activity as a complex. Some discrepancies were observed on the RNA level, which may explain the results of study showing that inactivation SSRP1 or SPT16 in tumor cells affected expression of slightly different sets of genes [9]. RNA levels of both FACT subunits were much less variable than protein levels. This may be a reflection of different sensitivities of the detection methods used and/or differences in the population of cells analyzed by each method (individual cells in IHC versus a pooled population in most RNA expression profiling studies). Alternatively, this may indicate regulation of FACT subunit abundance on the level of translation or protein stability, rather than (or in addition to) on the level of gene expression. In addition, conclusions about RNA levels were made based on analysis of multiple independent studies run under different conditions, which to some extent adds value to these observations. However, substantially different results coming from different experiments cannot just be averaged and may require investigation of the material and methods used in each study, which was not taken into account in our study.

FACT protein levels were variable between tissues and mosaic in those organs where FACT expression was detected. Most adult tissues did not have detectable levels of either FACT subunits. Positive cells were found in organs in which presence of proliferating or undifferentiated cells was expected with some exceptions such as neurons in brain or Langerhans islets in pancreas. The function of FACT in these cells remains to be investigated.

Hematopoietic (including lymphoid) and reproductive organs and crypts of intestine were the organs found to have the highest proportion of FACT-positive cells and one of the highest level of proliferative cells among adult tissues. The first two types of organs also showed the highest levels of FACT mRNA expression, while intestine was not FACT-positive in human or mouse mRNA expression studies. This may be due to the low proportion of FACT-positive cells among other cells in the intestine.

Mosaic expression of FACT among cells of the same type suggests that it may be associated with a particular cellular state. Analysis of in vivo distribution of the proliferative marker Ki67 and in vitro manipulations of cell proliferation demonstrated that FACT expression is not directly associated with the proliferative activity of cells. In addition, FACT levels were not changed during cell cycle transit [7]. A second potential explanation for the observed mosaic expression of FACT is its association with a certain stage of cell differentiation. Our finding of FACT expression specifically in the cells at the very bottom of intestinal crypts, but not those higher up in the crypt suggests that FACT may be expressed in undifferentiated progenitor cells or stem cells. This hypothesis is well in line with the expression profiling data for both FACT subunits: high expression levels were most frequently observed in different embryonic cells and fetal tissues and FACT mRNA levels were almost always higher in stem and progenitor cells as compared to differentiated cells. Moreover, this hypothesis was confirmed directly by our demonstration that induction of mouse C2C12 myoblast differentiation in vitro was accompanied by a sharp reduction in SPT16 and a gradual decrease in SSRP1 levels (Fig.5A). Importantly, the protocol for in vitro differentiation of C2C12 cells requires growth of the cells to high density before addition of differentiation medium. Cells at this density undergo growth arrest due to contact inhibition, which allowed us to measure FACT levels in growth arrested undifferentiated cells as compared to differentiated cells. FACT levels remained high in growth arrested cells and only dropped after induction of differentiation, thus confirming association of FACT expression with differentiation status rather than proliferative status.

Therefore, three types of data, distribution of FACT proteins in cells of different organs, expression profiles of FACT mRNAs in different experimental conditions, and direct experimental manipulations with cell differentiation and proliferation, all suggest that high FACT expression is a marker of undifferentiated, progenitor and stem cells and that low FACT expression is a marker of differentiated cells. Accumulated data from a number of different studies has revealed a so called embryonic stem (ES) cell-like gene expression signature [20]. SPT16 expression was already included in this signature based on finding of SPT16 among myc-responsive targets using microarray hybridization and myc binding to SPT16 promoter using ChIP-sequencing [20]. Our results confirm inclusion of SPT16 in an ES cell-like gene expression signature and indicate that SSRP1 should be included as well. Future studies will be required to establish the functional role of FACT in stem/progenitor cells and its effect on differentiation and dedifferentiation.

Association of FACT with ES cell-like signature explains potential role of FACT in cancer, as gene expression studies demonstrated presence of this signature in many cancers as well as other features of stem cells. From the other side, limited expression of FACT in adult differentiated cells and tissues suggests that FACT may be used as a target for temporal therapeutic inhibition in cancer treatment with limited harm to normal tissues.

## ACKNOWLEDGMENTS

Acknowledgements: We are grateful to Andrei Gudkov for critical reading and discussion of the manuscript. We also thank Patricia Stanhope Baker for help with manuscript preparation. This work was supported in part by grants from from Incuron, Inc. to K.V.G.

## MATERIAL AND METHODS

### Cells, constructs and chemicals

HT1080 cells were obtained from ATCC and maintained in DMEM supplemented with 10% heat inactivated (HI) FBS and antibiotics. HT1080-p21 cells were obtained from Igor Roninson (Department of Molecular Genetics, University of Illinois at Chicago) and were already described [21]. C2C12 skeletal myoblasts were obtained from Dr. Asoke Mal (Roswell Park Cancer Institute (RPCI), Buffalo, NY). Cells were maintained in growth medium consisting of DMEM with 20% HI FBS and antibiotics.

To induce differentiation of C2C12 cells, cells were transferred to differentiation medium (DM) consisting of DMEM with 2% HI horse serum (Gibco) and 10μg/mL insulin [13]. The formation of differentiated myotubes was observed within 24h of transfer to DM. Samples were collected for immunoblotting every 24h for 7d after transfer to DM. IPTG, propidium iodide and polybrene were from Sigma Aldrich, Inc. Lipofectamine 2000 Reagent was from Invitrogen. Lentivirus packaging and transduction was done as previously described [22].

### Immunoblotting

Cells were lysed in Cell Culture Lysis Reagent (Promega) and loaded on precast 4-20% gradient gels (Bio-Rad). Gels were blotted onto PVDF membranes (Bio-Rad, Inc.) and probed with the following antibodies: SPT16, SSRP1 (both from Biolegend, Inc.), anti-myosin heavy chain (MF20, kindly provided by Dr. Asoke Mal, Roswell Park Cancer Institute), and β-actin (Sigma-Aldrich, Inc.) as a loading control.

### Immunohistochemical staining

Sections of paraffin-embedded tissues were cut at 5μm, placed on charged slides, and dried at 60°C for one hour. Slides were cooled to room temperature, deparaffinized in three changes of xylene, and rehydrated using graded alcohols. Endogenous peroxidase was quenched with aqueous 3% H^2^O^2^. For antigen retrieval, slides were heated in citrate buffer (pH 6.0) in a microwave for 20 min , then cooled for 15 min and washed in PBS/T. Slides were then loaded onto a Dako Autostainer and blocked for 5 min with serum-free protein block (Dako). Blocked slides were then stained for 1 h with mouse monoclonal anti-SSRP1 (Biolegend; used at 1.7 μg/ml on human sections), goat polyclonal anti-SSRP1 (Santa Cruz, Cat. #sc-5909; used at 0.2 μg/ml on mouse sections), or goat polyclonal anti-SPT16 (Santa-Cruz, Cat. #sc-5915; used at 0.2 μg/ml on human and mouse sections). Isotype-matched control antibodies (1.7 μg/ml mouse IgG2b or 0.2 μg/ml goat IgG) were used on duplicate slides in place of the primary antibody as a negative control. After washing, slides were incubated with biotinylated goat anti-mouse IgG (Jackson ImmunoResearch Laboratories, Inc.) or donkey anti-goat IgG (Jackson ImmunoResearch Laboratories, Inc.), followed by the Elite ABC Kit (Vectastain), and DAB chromagen (Dako). Slides were then counterstained with Hematoxylin, dehydrated, cleared and covered with coverslips. All slides were scanned using Aperio system (Aperio Technologies, Inc). Images were made using Image scope software (Aperio Technologies, Inc).

### Animal experiments

All animal procedures were done according to a protocol approved by the RPCI IACUC. FVB mice were purchased from Taconic. Female and male 8 week-old animals were euthanized by CO^2^ inhalation. Immediately after sacrifice, organs were collected and fixed in 10% buffered formalin.

### Analysis of GEO datasets for FACT subunit mRNA expression

The NCBI GEO Profiles search engine [11, 12] was used to obtain all data entries in the NCBI Gene Expression Omnibus (GEO) database containing mammalian SSRP1 or SPT16 (Supt16h) gene expression analysis. From these, we selected all entries in which expression of SSRP1 or SPT16 was changed. The criterion for selection at this stage was a change in FACT subunit expression of 1.25-fold or more between any of the conditions within the experiment if the Student test p value was <0.05. If a p value was not available (no replicates), then a 2.0-fold change in expression was required for selection. The conditions that were identified as associated with changed SSRP1 or SPT16 expression were classified according to the biological process involved (see Table 2). Only categories with 2 or more independent experiments were used for further analysis.

The next step in our analysis involved identification of other experiments within each category of interest that measured SSRP1 or SPT16 expression, but did not show a change in expression allowing selection in the preceding step. This was done by performing GEO Profiles searches using word combinations of either SSRP1 or SPT16 and keywords for the biological process(es) included in each category (see Table 2 for the lists of categories and keywords). For some categories it was difficult to generate a keyword list sufficient for identification of all potential experiments (e.g., “Oncogenes activity”). In these cases, all mammalian SSRP1/ SPT16 entries were searched manually to find similar experiments (e.g., including all potential oncogenes, even if the word oncogene was not used in the experiment description).

Once all potential entries had been identified for all categories, we calculated (i) the percentage of experiments in a given category in which SSRP1 or SPT16 expression was changed; (ii) the maximal change in SSRP1 and/or SPT16 expression level within each category; and (iii) the average change in expression within each category (including all experiments in which FACT subunit mRNA levels were measured).

To identify categories in which FACT subunit expression was changed more frequently and/or more significantly than the “baseline” observed in all experiments, we selected data for two additional categories: “all experiments” and “experiments with knockout (KO) or knockdown (KD) of any gene”. In the first category, we included all entries in which expression of SSRP1 or SPT16 was measured in mammalian samples. In the second category, we included experiments in which keywords KO or KD were used. The proportion of experiments in which SSRP1 or SPT16 expression levels were changed within these two categories, as well as the average and maximal change (only for KO and KD experiment), was used to establish cut-off lines to distinguish experiments in which FACT expression changed with higher than background frequency and/or extent.We also calculated coefficient of correlation between change in SSRP1 level and SPT16 level for all three parameters (Pearson coefficient).

## Supplementary Figures and Tables














