# Supplementary figures and images for: Expression of FACT in mammalian tissues suggests its role in maintaining of undifferentiated state of cells
Source: Oncotarget. 2011 Oct 13;2(10):783–96. doi: 10.18632/oncotarget.340 (PMC3248156; doi:10.18632/oncotarget.340)

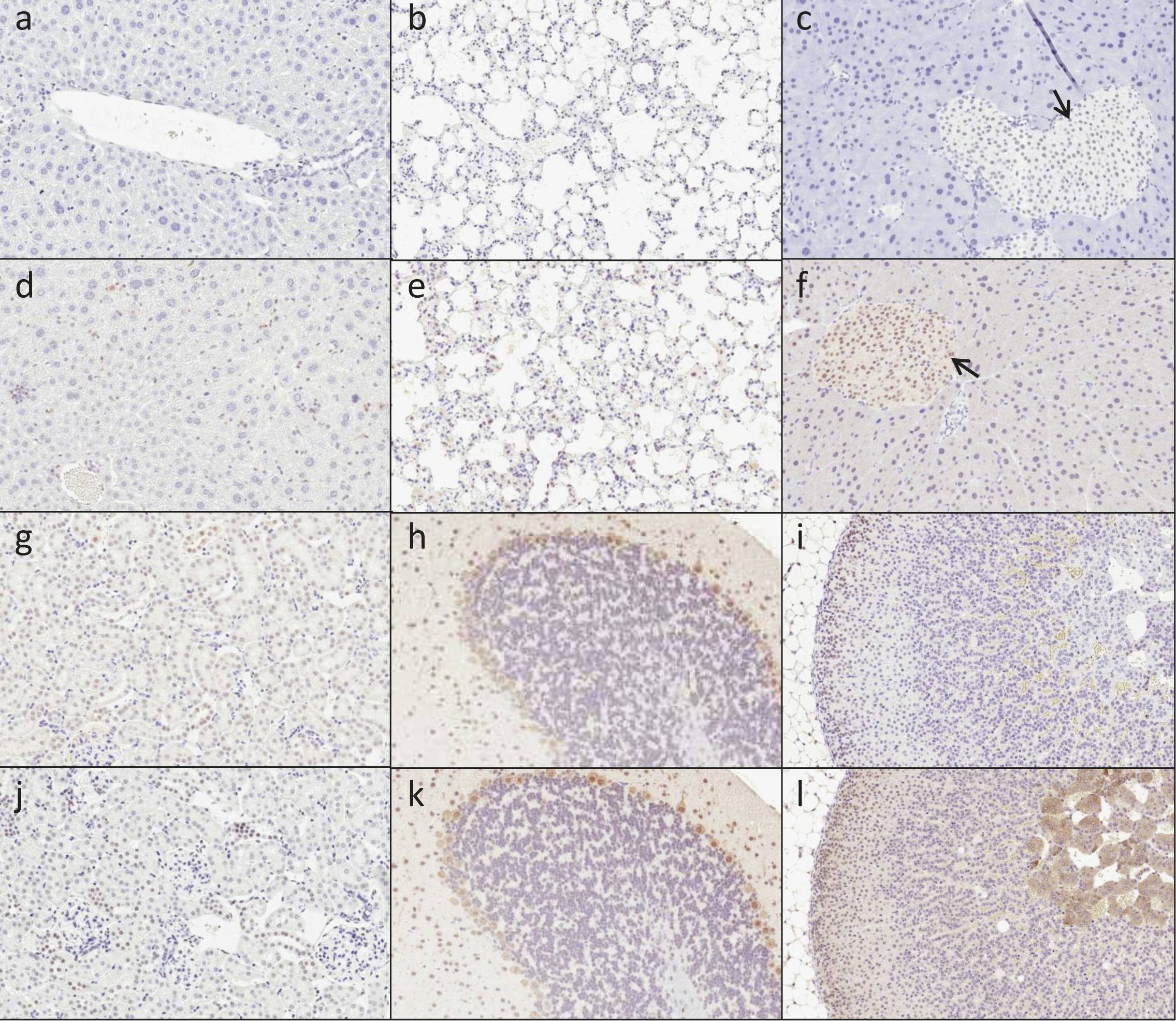

Supplement: Supplementary file 1 [file oncotarget-02-783-s001.tif]

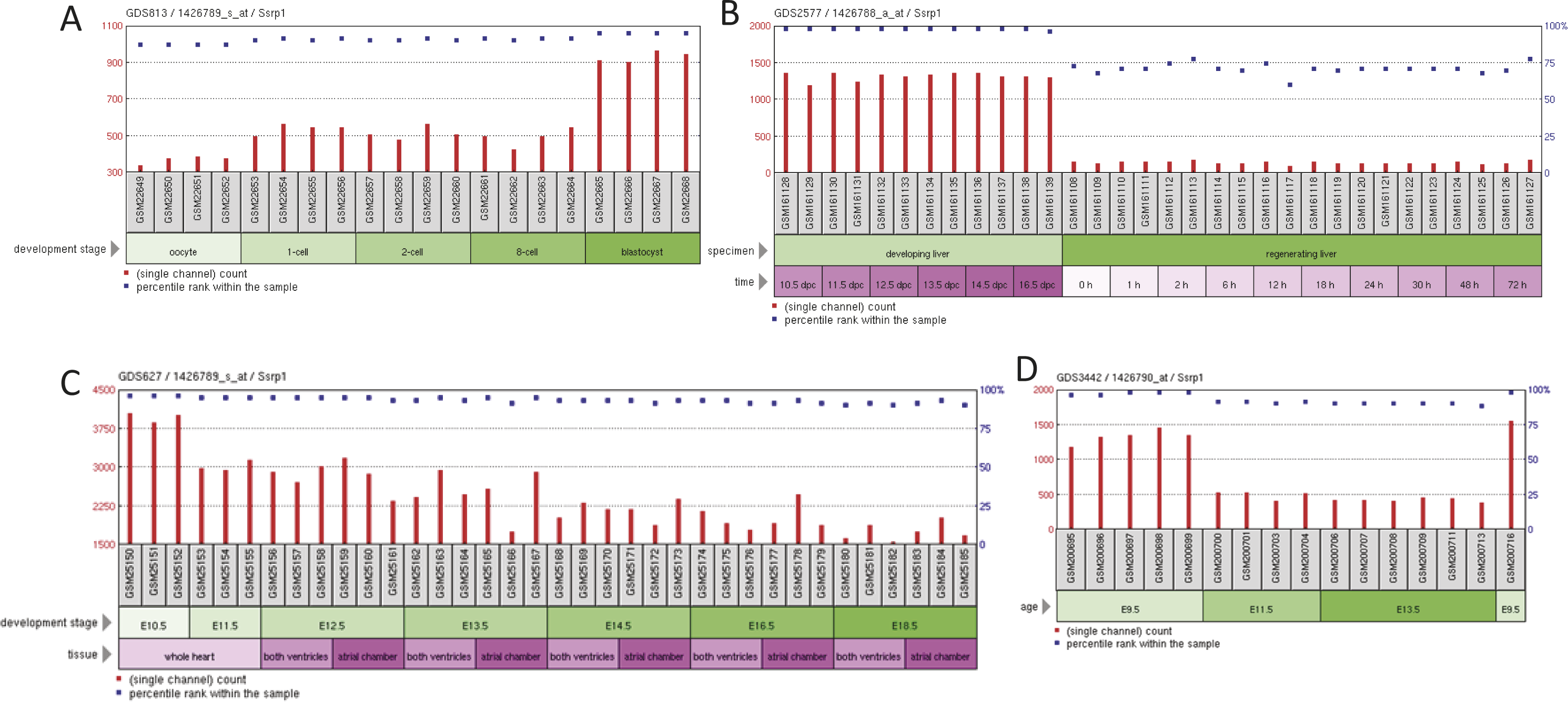

Supplement: Supplementary file 2 [file oncotarget-02-783-s002.tif]

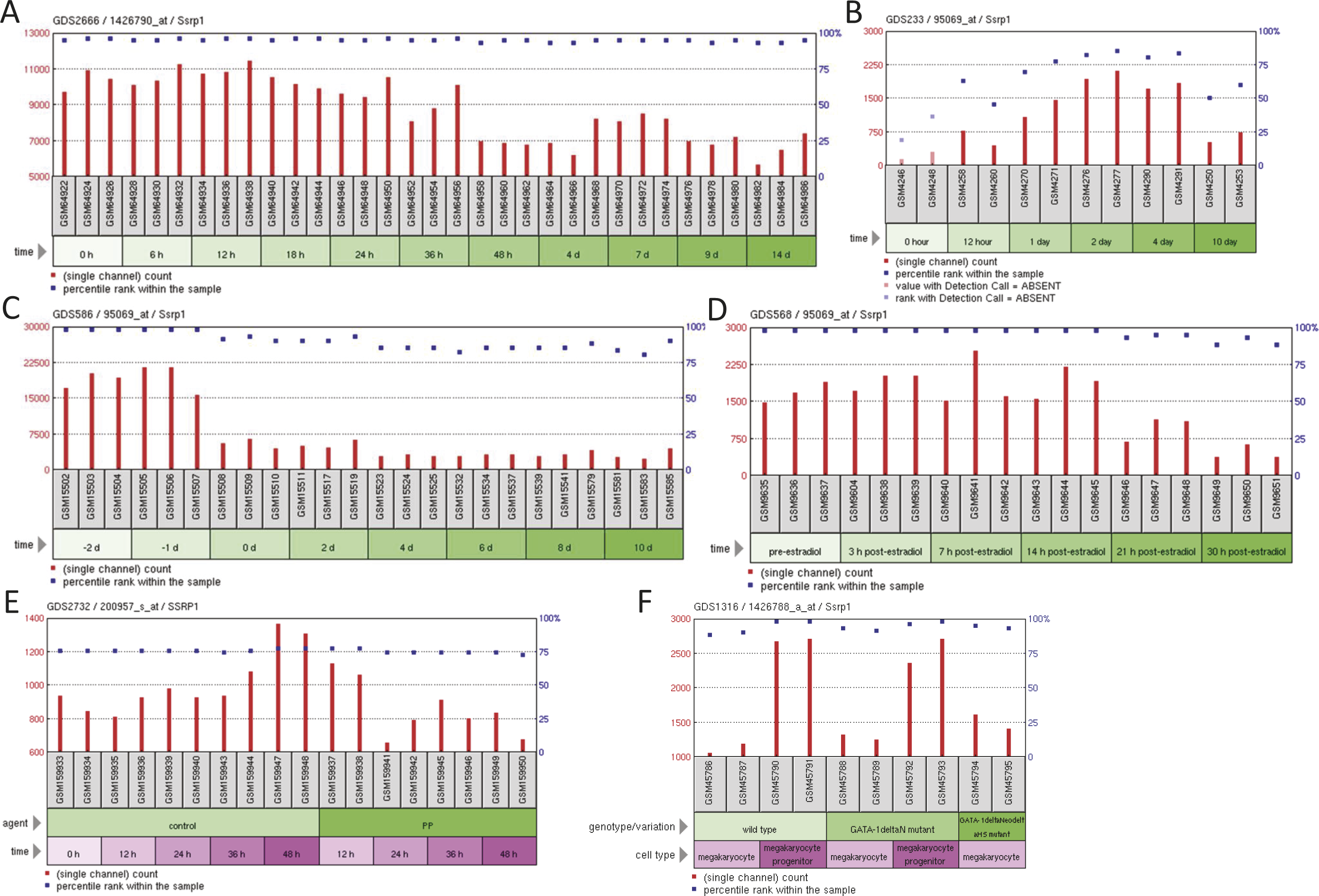

Supplement: Supplementary file 3 [file oncotarget-02-783-s003.tif]
