# Supplementary material for: Expression of FACT in mammalian tissues suggests its role in maintaining of undifferentiated state of cells
Source: Oncotarget. 2011 Oct 13;2(10):783–96. doi: 10.18632/oncotarget.340 (PMC3248156; doi:10.18632/oncotarget.340)
Supplement: Supplementary file 4 [file oncotarget-02-783-s004.docx]

Table S1. Previous studies analyzing expression of FACT subunit mRNAs in different normal organs of mouse and human.

| **GEO study number** | **species** | **number of tissues analyzed** | **high expression of SSRP1*** | **high expression of SPT16*** |
| --- | --- | --- | --- | --- |
| GDS181 | *H.sapiens* | 20 | uterus, testis, ovary, trachea | NA |
| GDS422,423 | *H.sapiens* | 12 | thymus | thymus |
| GDS596 | *H.sapiens* | 79 | placenta, uterus, testis | skeletal muscles, heart testis |
| GSD1085 | *H.sapiens* | 35 | testis, bladder | testis, bladder |
| GDS1096 | *H.sapiens* | 36 | bone marrow | no significant difference |
| GDS3113 | *H.sapiens* | 28 | thymus, ovary, uterus, tonsils | ovary, testis, peripheral blood lymphocytes, mammary gland |
| GDS565 | *M.musculus* | 5 | testis, ovary | testis, ovary, hypothalamus |
| GDS592 | *M.musculus* | 61 | mammary gland, ovary, umblical cord, uterus, lymph node, bone, bone marrow, thymus, trachea | NA |
| GDS1490 | *M.musculus* | 24 | spleen, testis, thymus | NA |
| GDS3142 | *M.musculus* | 22 | spleen, ovary, testis, mammary gland | spleen, ovary, testis |

NA – data are not available

* - 2 folds higher level is found in these tissues than mean of all tissues analyzed (p<0.05).
