# Supplementary material for: Expression of FACT in mammalian tissues suggests its role in maintaining of undifferentiated state of cells
Source: Oncotarget. 2011 Oct 13;2(10):783–96. doi: 10.18632/oncotarget.340 (PMC3248156; doi:10.18632/oncotarget.340)
Supplement: Supplementary file 5 [file oncotarget-02-783-s005.docx]

| **Table S2. Summary of studies, in which levels of FACT subunits were measured in cells at different stages of differentiation. In red – description of data showing increase of FACT levels with differentiation or decrease upon dedifferentiation.** | | | | | | | |
| --- | --- | --- | --- | --- | --- | --- | --- |
|  |  |  |  |  |  |  |  |
| **GEO Dataset Study** | **Description of experiment** | **SSRP1** | | | **SPT16 (=Supt16h)** | | |
|  |  | **description of change** | **fold change** | **p-value** | **description of change** | **fold change** | **p-value** |
| [GDS1010](http://www.ncbi.nlm.nih.gov/sites/GDSbrowser?acc=GDS1010) | Expression profiling of Side Population (SP) cells from bone marrow and muscle of 4 to 5 week old C57BL/10SnJ males. Main Population from bone marrow and muscle also examined. | increased in side versus main population | 2.50 | 0.0009 | No data | | |
| [GDS1095](http://www.ncbi.nlm.nih.gov/sites/GDSbrowser?acc=GDS1095) | Expression profiling of Lin-CD34-, Lin-CD34+, and Lin+CD34+ peripheral blood hematopoeitic stem cells (HSCs). Results provide insight into the molecular basis of the association of CD34 expression with self-renewal and lineage commitment in HSCs. | lowest in Lin- and CD34- | 1.64 | 0.05 | No data | | |
| [GDS1265](http://www.ncbi.nlm.nih.gov/sites/GDSbrowser?acc=GDS1265) | Analysis of oocytes from follicles at the primordial to large antral stages of development, collected from B6SJLF1 animals at 2 to 22 days of age. | reduction with differentiation | 1.54 | 0.000001 | reduction with differentiation | 2.60 | 0.000002 |
| [GDS1288](http://www.ncbi.nlm.nih.gov/sites/GDSbrowser?acc=GDS1288) | Mesenchymal stem cells (MSC) and undifferentiated embryonic stem cells (ESC). ESC plated on a monolayer of OP9 stromal cells in the presence of 20% heat inactivated fetal bovine serum in alpha MEM medium to induce differentiation into MSC. | the highest in undifferentiated ES | 2.71 | 0.0004 | the highest in undifferentiated ES | 2.04 | 0.003 |
| [GDS1316](http://www.ncbi.nlm.nih.gov/sites/GDSbrowser?acc=GDS1316) | Megakaryocytes and their progenitors from mutant E12.5 embryo liver expressing truncated transcription factor GATA-1. | higher in progenitors than in differentiated cells | 2.60 | 0.0011 | higher in progenitors than in differentiated cells | 1.68 | p<0.001 |
| [GDS1347](http://www.ncbi.nlm.nih.gov/sites/GDSbrowser?acc=GDS1347) | Comparison of mesenchymal stem cells (MSC) treated with dimethylsulphoxide and butylated hydroxyanisole (DMSO/BHA) for 6 or 48 hours to fetal brain (positive control) or adult liver (negative control). | reduction with differentiation | variable | - | reduction with differentiation | 1.5 | 0.046 |
| [GDS1396](http://www.ncbi.nlm.nih.gov/sites/GDSbrowser?acc=GDS1396) | Multipotent neural progenitor cells (NPCs) propagated by growth on media containing cystatin C and fibroblast growth factor-2. NPCs isolated from hippocampus neural stem cells. | reduction with differentiation | 1.60 | 1.69E-05 | No data | | |
| [GDS1475](http://www.ncbi.nlm.nih.gov/sites/GDSbrowser?acc=GDS1475) | Analysis of keratinocytes expressing activated Rho or Citron kinase (CRIK), or treated with C3 transferase. Rho plays a positive role during early differentiation, CRIK is a Rho effector, and C3 is a Rho inhibitor. | reduced in cells with activated Rho, but not Crik | 2.70 | 0.0008 | No data | | |
| [GDS1765](http://www.ncbi.nlm.nih.gov/sites/GDSbrowser?acc=GDS1765) | cell lines derived from extraocular (EOM) and gastrocnemius skeletal muscle allotypes at various time points up to 48 hours following induction of myogenesis. Results provide insight into the early developmental mechanisms underlying the formation of skeletal muscle classes. | big variability | variable | - | big variability | variable | - |
| [GDS1802](http://www.ncbi.nlm.nih.gov/sites/GDSbrowser?acc=GDS1802) | Colonic epithelial cells treated with sodium butyrate. Gene expression examined at various time points up to 24 hours following treatment. | reduction with differentiation | 2.30 | p<0.001 | not significant | - | - |
| [GDS1865](http://www.ncbi.nlm.nih.gov/sites/GDSbrowser?acc=GDS1865) | Micromass cultures derived from limb bud mesenchymal cells and cultured for 15 days in differentiating conditions (beta-glycerophosphate and ascorbic acid). | reduction with differentiation | 1.75 | 0.004 | reduction with differentiation | 1.85 | 0.002 |
| [GDS1882](http://www.ncbi.nlm.nih.gov/sites/GDSbrowser?acc=GDS1882) | CGR8 embryoid bodies differentiating for 3 or 10 days. Culturing of most embryonic stem (ES) cells require embryonic fibroblast (MEF) feeder cells which may affect their gene expression profiles. | reduction with differentiation (not significant) | - | - | not significant | - | - |
| [GDS2008](http://www.ncbi.nlm.nih.gov/sites/GDSbrowser?acc=GDS2008) | Umbilical vein endothelial cells (HUVEC) at various time points up to 48 hours following transfection with a recombinant adenovirus expressing early growth response-1 (EGR1). EGR1 is implicated in cell growth, apoptosis, and differentiation. | reduction with differentiation | 1.80 | p<0.1 | not significant | - | - |
| [GDS2151](http://www.ncbi.nlm.nih.gov/sites/GDSbrowser?acc=GDS2151) | C2C12 skeletal muscle cells following shRNA knockdown of the RNA helicases p68 and p72. C2C12 myoblasts cultured in differentiation medium to induce differentiation into skeletal muscle cells. | reduction with differentiation | 2.60 | 0.0002 | reduction with differentiation | 1.63 | 0.007 |
| [GDS2227](http://www.ncbi.nlm.nih.gov/sites/GDSbrowser?acc=GDS2227) | Comparison of cells induced to differentiate into dopaminergic neurons by guided differentiation (GD) to those induced to differentiate into embryoid bodies (EBs) by random differentiation (RD). Cells at 3 stages of differentiation in the GD model and days 4 to 21 EBs in the RD model compared. | reduction with differentiation | 2.00 | 0.003 | not significant | 1.81 | 0.27 |
| [GDS2276](http://www.ncbi.nlm.nih.gov/sites/GDSbrowser?acc=GDS2276) | Analysis of transgenic embryonic stem cells induced with doxycycline to express neurogenin 3 (Ngn3) and differentiated for 3 or 10 days as embryoid bodies. Ngn3 is required for pancreatic islet development. | reduction with differentiation | 1.72 | 0.0004 | not significant | - | 0.27 |
| [GDS2276](http://www.ncbi.nlm.nih.gov/sites/GDSbrowser?acc=GDS2276) | Analysis of transgenic embryonic stem cells induced with doxycycline to express neurogenin 3 (Ngn3) and differentiated for 3 or 10 days as embryoid bodies. Ngn3 is required for pancreatic islet development. | reduction with differentiation | 1.25 | 0.001323 | reduced in Ainv15 embryonic bodies comparing with undifferentiated ES cells | 2.14 | 0.002149 |
| [GDS2322](http://www.ncbi.nlm.nih.gov/sites/GDSbrowser?acc=GDS2322) | Analysis of VEGF receptor Flk1-expressing cells isolated from embryoid bodies up to 8 days post-differentiation. | reduction with differentiation | 2.30 | p<0.001 | reduction with differentiation | 3.57 | p<0.001 |
| [GDS233](http://www.ncbi.nlm.nih.gov/sites/GDSbrowser?acc=GDS233) | Cardiotoxin injected into mouse gastrocnemius muscle to induce muscle regeneration. Muscles profiled at 27 time points (0-40 days) post-injection. | elevated in regenerated muscle | 8.56 | 0.005 | No data | | |
| [GDS2375](http://www.ncbi.nlm.nih.gov/sites/GDSbrowser?acc=GDS2375) | Analysis of 5 embryonic stem cell (ESC) lines. Results provide insight into the molecular mechanisms that maintain ESCs in a pluripotent state, and the differences between human and non-human primate ESCs. | the level is higher in embryonic stem cells than in embrionic bodies than in embryonic fibroblasts | 4.59 | - | higher in ESC than fibroblasts than embryoid bodies | 1.88 | - |
| [GDS2395](http://www.ncbi.nlm.nih.gov/sites/GDSbrowser?acc=GDS2395) | Analysis of cDNA libraries each generated from a single cultured epidermal stem cell (SC) or transit-amplifying (TA) cell. TA cells are SC progenies that are destined to terminally differentiate. | lower in TA cells than in stem cells | variable | - | no change | 1.0 | - |
| [GDS2398](http://www.ncbi.nlm.nih.gov/sites/GDSbrowser?acc=GDS2398) | Analysis of hematopoietic stem cells (HSCs), progenitor cells (HPCs), and granulocytes. Short-term (ST) and long-term (LT) repopulating HSCs examined. | reduction with differentiation | 6.26 | p<0.01 | reduction with differentiation | 4.92 | p<0.01 |
| [GDS2412](http://www.ncbi.nlm.nih.gov/sites/GDSbrowser?acc=GDS2412) | Analysis of C2C12 skeletal myoblasts induced to differentiate into myotubes in vitro. Results provide insight into the role of nuclear envelope transmembrane proteins in myogenesis. | reduction with differentiation | 1.88 | 0.000694 | reduction with differentiation | 1.80 | 0.001874 |
| [GDS2420](http://www.ncbi.nlm.nih.gov/sites/GDSbrowser?acc=GDS2420) | Mock and deltaNp73alpha-expressing C2C12 myoblasts 6 and 24 hours after shifting the cells to a differentiation medium. DeltaNp73alpha, a p73alpha isoform, functions as a transdominant inhibitor of other p53 family members, interfering with multiple developmental programs. | reduction with differentiation in mock but not in deltaNp73alpha | 2.10 | .- | reduction with differentiation in mock but not in deltaNp73alpha | 1.87 | - |
| [GDS2421](http://www.ncbi.nlm.nih.gov/sites/GDSbrowser?acc=GDS2421) | Analysis embryonic fibroblasts after treatment with fibroblast growth factor 2 (FGF2) at 4, 13, and 40 ng/ml for 24 hours. FGF2 promotes the self-renewal of embryonic stem cells . | increase upon FGF2 treatment | 1.50 | - | increase upon FGF2 treatment | 1.34 | - |
| [GDS2422](http://www.ncbi.nlm.nih.gov/sites/GDSbrowser?acc=GDS2422) | Analysis of embryonic fibroblasts starved of fibroblast growth factor 2 (FGF2) for 2 days and subsequently restimulated with FGF2. FGF2 promotes the self-renewal of embryonic stem cells. | higher in starved than in restimulated | 1.08 | 0.1 | higher in starved than in restimulated | 1.21 | 0.35 |
| [GDS2423](http://www.ncbi.nlm.nih.gov/sites/GDSbrowser?acc=GDS2423) | Analysis of embryonic fibroblast-like (EF) differentiated cells derived from H9 embryonic stem cells. Foreskin fibroblasts, and undifferentiated H9 and embryonic carcinoma cells also examined. | reduction with differentiation | 2.72 | 0.056 | reduction with differentiation | 2.69 | 0.147 |
| [GDS2429](http://www.ncbi.nlm.nih.gov/sites/GDSbrowser?acc=GDS2429) | monocytes (MCs) treated with M-CSF to induce differentiation into macrophages (MPs), and mature MPs treated with either IFN-gamma and LPS or IL-4 to induce polarization to M1 or M2 cells, respectively. | reduced with differentiation and then elevated in M1 and M2 | variable | - | reduced with differentiation and then elevated in M1 and M2 | variable | - |
| [GDS2431](http://www.ncbi.nlm.nih.gov/sites/GDSbrowser?acc=GDS2431) | Analysis of adult differentiating CD34+ hematopoietic progenitor cells at various time points up to 11 days of growth in serum-free medium containing erythropoietin, interleukin-3 and stem cell factor. | bell shape | 1.60 | - | not significant | - | - |
| [GDS2440](http://www.ncbi.nlm.nih.gov/sites/GDSbrowser?acc=GDS2440) | Analysis of dendritic cell (DC) development from Flt3(+)CD11b(+) DC progenitors. DC progenitors treated with GM-CSF for 7 and 10 days to induce differentiation. DCs differentiated for 10 days were subsequently treated with TNFalpha to induce maturation. | reduction with differentiation | 4.26 | - | No data | | |
| [GDS2521](http://www.ncbi.nlm.nih.gov/sites/GDSbrowser?acc=GDS2521) | Analysis of primary megakaryocytes (MK) purified after 3 to 6 days in culture. MKs undergo sequential morphologic and functional transitions that culminate in platelet assembly in proplatelets. | reduction with differentiation | 2.10 | .01>p>.001 | reduction with differentiation | 2.33 | .05>p>.01 |
| [GDS2660](http://www.ncbi.nlm.nih.gov/sites/GDSbrowser?acc=GDS2660) | differentiating 3T3-L1 preadipocytes at various time points up to 28 days after induction of adipogenesis using a standard differentiation cocktail of methylisobutylxanthine, dexamethasone, and insulin (MDI). | bell shape | 2.10 | - | No data | | |
| [GDS2666](http://www.ncbi.nlm.nih.gov/sites/GDSbrowser?acc=GDS2666) | Analysis of R1 embryonic stem cells differentiating into embryoid bodies in vitro. Cells examined at various time points up to 14 days after inducing differentiation. | reduction with differentiation | 1.90 | .01>p>.001 | reduction with differentiation | 1.84 | 0.0036 |
| [GDS2668](http://www.ncbi.nlm.nih.gov/sites/GDSbrowser?acc=GDS2668) | Analysis of J1 embryonic stem cells differentiating into embryoid bodies in vitro. Cells examined at various time points up to 14 days after inducing differentiation. | reduction with differentiation | 1.51 | 0.006 | reduction with differentiation | 1.89 | 0.00086 |
| [GDS2671](http://www.ncbi.nlm.nih.gov/sites/GDSbrowser?acc=GDS2671) | Analysis of V6.5 embryonic stem cells differentiating into embryoid bodies in vitro. Cells examined at various time points up to 14 days after inducing differentiation. | reduction with differentiation | 1.44 | 0.007 | reduction with differentiation | 2.33 | 0.00059 |
| [GDS2725](http://www.ncbi.nlm.nih.gov/sites/GDSbrowser?acc=GDS2725) | Analsysis of conditionally immortalized astrocyte RCG-12 cells induced to undergo growth arrest and differentiation by heat inactivation of a temperature-sensitive large T-antigen. | reduction with differentiation | 1.21 | 0.072 | not significant | 1.12 | 0.29 |
| [GDS2732](http://www.ncbi.nlm.nih.gov/sites/GDSbrowser?acc=GDS2732) | Epidermal keratinocytes at various time points up to 48 hours following treatment with 2-(3,4,5-trimethoxyphenylamino)-pyrrolo[2,3-d]pyrimidine (PP). PP induces the terminal differentiation of epidermal keratinocytes. | increased in control and decreased after treatment with PP | 2.40 | p<0.05 | reduction with differentiation | 1.61 | 0.002 |
| [GDS2743](http://www.ncbi.nlm.nih.gov/sites/GDSbrowser?acc=GDS2743) | Brown and white preadipocytes at the undifferentiated and differentiating stages. Unlike white adipocytes, brown adipocytes have an abundance of mitochondria and are thus able to contribute to energy expenditure. | reduction with differentiation, but only of brown adipocytes | 1.91 | 0.0001 | No data | | |
| [GDS2810](http://www.ncbi.nlm.nih.gov/sites/GDSbrowser?acc=GDS2810) | Analysis of two nonmalignant mammary epithelial cells (HMEC) grown in a laminin-rich extracellular matrix. Both HMECs transit from a disorganized to an organized state to form polarized acini. | reduction with time | 1.30 | p<0.05 | reduction with time | 1.69 | 0.001614 |
| [GDS2926](http://www.ncbi.nlm.nih.gov/sites/GDSbrowser?acc=GDS2926) | Analysis of phorbol ester-treated CHRF-288-11 megakaryoblastic cells induced to undergo megakaryocytic (Mk) differentiation and primary Mk (PriMk) cells derived from cytokine-treated CD34+ peripheral blood cells. | reduction with differentiation | 2.10 | 0.0278 | reduction with differentiation | 3.33 | 0.0026 |
| [GDS2940](http://www.ncbi.nlm.nih.gov/sites/GDSbrowser?acc=GDS2940) | Analysis of dendritic cells (DCs) at various time points up to 36 hours following treatment with TGF-beta1. DCs derived from CD34+ hematopoietic progenitor cells induced to differentiate in vitro. | reduction with differentiation | 2.87 | 4.01E-05 | No data | | |
| [GDS3002](http://www.ncbi.nlm.nih.gov/sites/GDSbrowser?acc=GDS3002) | MC3T3-E1 preosteoblasts treated with the histone deacetylase inhibitor (HDI) trichostatin A, MS-275, or valproic acid for 18 hours under osteogenic conditions. HDIs accelerate osteoblast maturation. | reduction upon TSA treatment | 1.59 | 3.97E-06 | reduction upon TSA treatment | 1.23 | p<.05 |
| [GDS3025](http://www.ncbi.nlm.nih.gov/sites/GDSbrowser?acc=GDS3025) | C3H10T1/2 (10T1/2) stem cells committed to the adipocyte lineage by treatment with bone morphogenetic protein 4 (BMP-4) | variable | - | - | slight decrease with differentiation | 1.1 | - |
| [GDS3032](http://www.ncbi.nlm.nih.gov/sites/GDSbrowser?acc=GDS3032) | Analysis of post-confluent Caco-2 colon cancer cells up to 10 days after treatment with ascorbate-stabilized quercetin, a polyphenol antioxidant compound. Caco-2 cells differentiate after reaching confluency. | reduction with differentiation | 1.47 | p<0.01 | not significant | 1.39 | 0.09 |
| [GDS3159](http://www.ncbi.nlm.nih.gov/sites/GDSbrowser?acc=GDS3159) | Analysis of C2C12 myoblast cells treated with reversine. Reversine induces dedifferentiation of C2C12 myoblasts and the subsequent redifferentiation to become multipotent mesenchymal progenitor cells. | reduction with **de**differentiation | 2.90 | 0.003 | reduction with differentiation | 2.69 | 6.09E-05 |
| [GDS3222](http://www.ncbi.nlm.nih.gov/sites/GDSbrowser?acc=GDS3222) | Analysis of cytotoxic T cell line (CTLL-2) at various time points up to 24 hours following interleukin-2 cytokine (IL-2) stimulation. IL-2 regulates T cell proliferation and differentiation. | increased with IL-2 treatment | 2.35 | 0.000003 | increased with IL-2 treatment, although not as significant as SSRP1 | 1.36 | p > 0.05 |
| [GDS3346](http://www.ncbi.nlm.nih.gov/sites/GDSbrowser?acc=GDS3346) | Analysis of a gastric epithelial progenitor (mGEP) cell line infected with two Helicobacter pylori strains: chronic atrophic gastritis (ChAG)-associated Kx1 and gastric cancer-associated Kx2 | higher in progenitors infected with with gastritis associated strain. No change in gastric cancer associated strain. | 1.30 | 0.00002 | no change | 1.00 | - |
| [GDS3405](http://www.ncbi.nlm.nih.gov/sites/GDSbrowser?acc=GDS3405) | primitive erythroid precursors and epithelial cells isolated from frozen sections of the embryonic day 9.5 yolk sac. | higher in erythroid precursors than in epithelial cells (not significant) | 1.43 | 0.087 | not significant | 1.57 | 0.13 |
| [GDS3513](http://www.ncbi.nlm.nih.gov/sites/GDSbrowser?acc=GDS3513) | Analysis of cardiomyocytes (CMs) derived from embryonic stem cells (ESCs). Under the appropriate conditions, ex vivo ESCs can differentiate into beating cardiomyocytes via an embryoid body (EB) intermediate. | reduction with differentiation | 2.10 | 0.04 | reduction with differentiation | 1.40 | p<0.001 |
| [GDS434](http://www.ncbi.nlm.nih.gov/sites/GDSbrowser?acc=GDS434) | Transcriptional profiling of embryonic stem (ES) cell lines with mutations affecting cholesterol transport and metabolism. Abca1 +/- and Cdk4 +/- ES lines compared with controls. | higher in ES cells than in liver | 5.66 | 0.001 | No data | | |
| [GDS51](http://www.ncbi.nlm.nih.gov/sites/GDSbrowser?acc=GDS51) | Differentiation of inner ear hair cell precursors in vitro. A conditionally immortal cell line derived from the mouse cochlea, UB/OC-1, examined from 0 to 14 days. | reduction with differentiation | variable | - | No data | | |
| [GDS568](http://www.ncbi.nlm.nih.gov/sites/GDSbrowser?acc=GDS568) | Analysis of erythroid differentiation using G1E ER4 clone cells. Estradiol addition induces Gata-1 triggering synchronous differentiation. 30 hour time course corresponds to late burst-forming unit-erythroid stage through orthochromatic erythroblast stage. | reduction with differentiation | 3.39 | 0.0005 | No data | | |
| [GDS586](http://www.ncbi.nlm.nih.gov/sites/GDSbrowser?acc=GDS586) | C2C12 myoblasts induced to differentiate and examined during cell proliferation (days -2 to -1), at cell cycle withdrawal (day 0) and during myogenic fusion and maturation of multinucleated myotubes (days 2 to 10). | reduction with differentiation | 3.80 | 0 | No data | | |
| [GDS587](http://www.ncbi.nlm.nih.gov/sites/GDSbrowser?acc=GDS587) | Analysis of early stages of myogenesis. C2C12 myoblasts induced to differentiate and examined during cell proliferation (days -2 to -1), at cell cycle withdrawal (day 0) and during myogenic fusion and maturation of multinucleated myotubes (days 2 to 10). | reduction with differentiation | 2.10 | no data | reduction with differentiation | 2.01 | 0.03 |
| [GDS687](http://www.ncbi.nlm.nih.gov/sites/GDSbrowser?acc=GDS687) | Dermal papilla (DP) induced epithelial stem cell differentiation. Keratinocyte stem cells from bulge area of telogen hair follicle co-cultured with DP over a 5 day period. | small reduction with diffferenatiation | 1.22 | - | No data | | |
| [GDS709](http://www.ncbi.nlm.nih.gov/sites/GDSbrowser?acc=GDS709) | Colonic adenocarcinoma cell line Caco-2 BBe as a model. Cells examined at 2, 8, and 15 days in culture: these are in the proliferating, postproliferative nondifferentiated, and differentiated stages respectively. | no difference | 1.08 | 0.0002 | No data | | |
| [GDS785](http://www.ncbi.nlm.nih.gov/sites/GDSbrowser?acc=GDS785) | CD4+ T cell differentiation by expression profiling of subpopulations of CD4+ cells representing 5 successive stages of differentiation: intrathymic T progenitors, double positive thymocytes, single positive thymocytes, naïve T cells from cord blood, and naïve T cells from adult blood. | reduction with differentiation | 1.90 | 0.0002 | reduction with differentiation | 1.6 | 0.022095 |
| [GDS799](http://www.ncbi.nlm.nih.gov/sites/GDSbrowser?acc=GDS799) | A404 cells at 48 and 96 hours following 1 umol/L all trans-retinoic acid (RA) treatment to induce differentiation into smooth muscle cells (SMCs). A set of cells treated with RA for 96 hours was subsequently treated with 0.5 ug/ml puromycin to enrich for SMCs. | big variability | variable | - | reduction with differentiation | 2.23 | p<0.000001 |
| [GDS826](http://www.ncbi.nlm.nih.gov/sites/GDSbrowser?acc=GDS826) | K562 erythroleukemia cells induced to differentiate into erythroid-like cells by hemin. K562 cells examined at various time points up to 72 hours following treatment with 50 uM hemin. | reduction with differentiation | 2.20 | 0.027 | not significant | 1.21 | 0.1 |
| [GDS853](http://www.ncbi.nlm.nih.gov/sites/GDSbrowser?acc=GDS853) | FACS-isolated erythroid progenitor and non-erythroid granulocyte-monocyte progenitor cells from 10-16 week old C57BL/6-S129Ola. | higher in progenitor cells | 1.50 | - | weakly higher in progenitor | 1.35 | - |
| [GDS890](http://www.ncbi.nlm.nih.gov/sites/GDSbrowser?acc=GDS890) | Expression profiling of neural crest stem cells at embryonic day (E) 9.5 and developing Schwann cells (SC) at E12, E14, E16, E18, and mature SCs at birth. Cells isolated by FACS from proteolipid-GFP transgenics. | reduction with differentiation | 3.26 | 0.0072 | No data | | |
| [GDS927](http://www.ncbi.nlm.nih.gov/sites/GDSbrowser?acc=GDS927) | Analysis of three distinct stages of late erythroid progenitor cell development. Developmentally synchronized erythroid progenitor cells (EPCs) were prepared from splenocytes at 80, 100 and 120 hours after thiamphenical withdrawal. | reduction with differentiation from 80 to 100 hrs | 4.16 | 0.025 | No data | | |
| [GDS970](http://www.ncbi.nlm.nih.gov/sites/GDSbrowser?acc=GDS970) | Temporal analysis of hepatoblast cell line 3 (HBC-3) cells grown on Matrigel to induce in vitro bile ductular differentiation. HBC-3 line is derived from liver diverticulum on day 9.5 of gestation. | reduction with differentiation | 1.54 | 0.004287 | not significant | 2.02 | 0.110288 |
