# Supplementary material for: Expression of FACT in mammalian tissues suggests its role in maintaining of undifferentiated state of cells
Source: Oncotarget. 2011 Oct 13;2(10):783–96. doi: 10.18632/oncotarget.340 (PMC3248156; doi:10.18632/oncotarget.340)
Supplement: Supplementary file 6 [file oncotarget-02-783-s006.docx]

| **Table S3. Summary of studies in which levels of FACT subunits were measured between cells of different proliferative status.** | | | | | |  |  |
| --- | --- | --- | --- | --- | --- | --- | --- |
|  |  |  |  |  |  |  |  |
| **GEO Dataset Study** | **Description of experiment** | **SSRP1** | | | **SPT16 (=Supt16h)** | | |
|  |  | **description of change** | **fold change** | **p-value** | **description of change** | **fold change** | **p-value** |
| [GDS911](http://www.ncbi.nlm.nih.gov/sites/GDSbrowser?acc=GDS911) | T98G cancer cells in growth-arrest induced by 3 days of either serum deprivation or contact inhibition | serum deprived condition < contact inhibited cells < in asynchronous cells | 3.69 | 0.0003 | lower in growth arrested cells | 1.96 | 0.015 |
| [GDS3089](http://www.ncbi.nlm.nih.gov/sites/GDSbrowser?acc=GDS3089) | HL60 treated with tretinoin (all-trans retinoic acid; ATRA) or vehicle. ATRA induces terminal differentiation or growth arrest in HL60 cells | reduction with growth arrest/differentiation | 3.31 | 0.000004 | reduction with growth arrest/differentiation | 2.69 | 0.0046592 |
| [GDS3062](http://www.ncbi.nlm.nih.gov/sites/GDSbrowser?acc=GDS3062) | Temporal analysis of cultures of multipotent stromal cells (MSCs) from bone marrow aspirates from 3 donors. On day 2, the MSCs are in transition from lag phase to log phase; on day 7, they are in stationary phase. Results provide insight into the molecular changes in MSCs during in vitro expansion. | reduction in stationary phase | 2.37 | 0.001 | reduction in stationary phase | 1.41 | 0.0027 |
| [GDS1802](http://www.ncbi.nlm.nih.gov/sites/GDSbrowser?acc=GDS1802) | Colonic epithelial cells treated with sodium butyrate | reduction with growth arrest | 2.27 | 0.0005 | NOT SIGNIFICANT | 1.30 | 0.4262 |
| [GDS3638](http://www.ncbi.nlm.nih.gov/sites/GDSbrowser?acc=GDS3638) | MDB-MB-453 breast cancer cells treated with 20 or 40 ug/ml actein for 6 or 24 hours. Actein is a triterpene glycoside from the herb black cohosh and inhibits the growth of cancer cells in vitro. | reduction after 24hr of 40ug/mL actein treatment | 2.14 | 0.0011 | reduction after 24hr of 40ug/mL actein treatment | 2.10 | 0.0008199 |
| [GDS2959](http://www.ncbi.nlm.nih.gov/sites/GDSbrowser?acc=GDS2959) | Analysis of leukocytes from individuals before and after the administration of granulocyte colony-stimulating factor (G-CSF). G-CSF is used to boost granulocyte counts in immunocompromised patients. | reduction after G-CSF treated cells | 2.05 | 0.000057 | reduction after G-CSF treated cells | 1.56 | 0.0004 |
| [GDS1518](http://www.ncbi.nlm.nih.gov/sites/GDSbrowser?acc=GDS1518) | Ureteric buds (UBs) cultured in the presence of non-branching growth factor heregulin (HRG) or branching growth factor pleiotrophin (PTN). | lower in heregulin treated cells than in other condtions | 1.78 | 0.004605 | lower in heregulin treated cells than in other condtions | 1.53 | 0.0401026 |
| [GDS2704](http://www.ncbi.nlm.nih.gov/sites/GDSbrowser?acc=GDS2704) | Smooth muscle cells treated for up to 480 minutes with platelet-derived growth factor (PDGF) | Higher in CHL1/Hey2 null cells treated with PDGF | 1.69 | - | Higher in CHL1/Hey2 null cells treated with PDGF | 1.59 | - |
| [GDS337](http://www.ncbi.nlm.nih.gov/sites/GDSbrowser?acc=GDS337) | Overexpression of the telomerase catalytic subunit (TERT) in mammary epithelial cells (HMEC). Findings imply that ectopic telomerase expression modulates growth-controlling genes and enhances cell proliferation. | higher in TERT expressing cells | 1.57 | 0.009515 | No data | | |
| [GDS3046](http://www.ncbi.nlm.nih.gov/sites/GDSbrowser?acc=GDS3046) | K562 treated with imatinib. | reduction with growth arrest | 1.56 | 0.0004 | reduction with growth arrest | 1.27 | 0.0181155 |
| [GDS2421](http://www.ncbi.nlm.nih.gov/sites/GDSbrowser?acc=GDS2421) | Embryonic fibroblasts after treatment with fibroblast growth factor 2 (FGF2) at 4, 13, and 40 ng/ml for 24 hours. FGF2 promotes the self-renewal of embryonic stem cells (ESCs) | higher in treated cells | 1.50 | - | higher in treated cells | 1.34 | - |
| [GDS3484](http://www.ncbi.nlm.nih.gov/sites/GDSbrowser?acc=GDS3484) | MCF-7 cells stimulated with insulin-like growth factor-I (IGF-I) for 3 or 24 hours | higher in treated cells | 1.49 | 0.00004 | NOT SIGNIFICANT | 1.15 | 0.3364129 |
| [GDS2810](http://www.ncbi.nlm.nih.gov/sites/GDSbrowser?acc=GDS2810) | Two nonmalignant mammary epithelial cells (HMEC) grown in a laminin-rich extracellular matrix. Both HMECs transit from a disorganized to an organized state to form polarized acini. | reduction with growth arrest | 1.35-immortal 1.46-finite | 0.0257-immortal 0.0327-finite | reduction with growth arrest | 1.69-immortal 1.48-finite | 0.0016-immortal .0069-finite |
| [GDS3148](http://www.ncbi.nlm.nih.gov/sites/GDSbrowser?acc=GDS3148) | hepatocytes treated with HGF for up to 24 hours. | higher in WT cells treated with HGF at 12hr | 1.37 | 0.019267 | NOT SIGNIFICANT | 1.41 | 0.1655949 |
| [GDS3344](http://www.ncbi.nlm.nih.gov/sites/GDSbrowser?acc=GDS3344) | Analysis of spontaneously immortalized embryonic fibroblasts (EFs) lacking the LIM-only protein FHL2. FHL2 deficiency results in reduced proliferative capacity of spontaneously immortalized EFs. | higher in WT cells | 1.34 | 0.0003 | higher in null cells | 2.22 | 0.002 |
| [GDS2725](http://www.ncbi.nlm.nih.gov/sites/GDSbrowser?acc=GDS2725) | Immortalized astrocyte RCG-12 cells induced to undergo growth arrest and differentiation by heat inactivation of a temperature-sensitive large T-antigen. | reduction with growth arrest | 1.21 | 0.072204 | NOT SIGNIFICANT | 1.12 | 0.2933847 |
| [GDS3267](http://www.ncbi.nlm.nih.gov/sites/GDSbrowser?acc=GDS3267) | MDCK cells exposed to hepatocyte growth factor (HGF), a MAPK pathway activator. MDCK cells also exposed to HGF plus MAPK inhibitors. Epithelial organs such as kidney develop via tubulogenesis, a process induced by MAPK activation. | variable, slightly lower levels with HGF treatment | 1.14 | 0.0001 | NO CHANGE | 1.00 | - |
| [GDS1736](http://www.ncbi.nlm.nih.gov/sites/GDSbrowser?acc=GDS1736) | Analysis of PC-3 prostate cancer cells incubated with arachidonic acid (AA). AA is an omega-6 fatty acid shown to induce cancer cell proliferation. | no change | 1.00 | - | NO CHANGE | 1.00 | - |
| [GDS2970](http://www.ncbi.nlm.nih.gov/sites/GDSbrowser?acc=GDS2970) | Leukemia K562 cells treated with the polyamide-chlorambucil conjugate 1R-Chl. 1R-Chl down-regulates transcription of the human histone H4c gene and inhibits the growth of several cancer cell lines in vitro. | no change | 1.00 | - | NO CHANGE | 1.00 | - |
| [GDS1922](http://www.ncbi.nlm.nih.gov/sites/GDSbrowser?acc=GDS1922) | Aortic rings treated with angiopoietin-1 (Ang-1) or vascular endothelial growth factor (VEGF). Aortic rings can generate neovessels ex vivo after angiogenic factor stimulation. | no change | 1.00 | - | NO CHANGE | 1.00 | - |
| [GDS2975](http://www.ncbi.nlm.nih.gov/sites/GDSbrowser?acc=GDS2975) | Immortalized ovarian surface epithelial (IOSE) cells treated for up to 12 hours with transforming growth factor beta 1 (TGFb1). IOSE cells are derived from normal ovarian epithelial cells which, unlike ovarian cancer, respond to TGFb1-induced growth inhibition. | no change | 1.00 | - | NO CHANGE | 1.00 | - |
| [GDS3568](http://www.ncbi.nlm.nih.gov/sites/GDSbrowser?acc=GDS3568) | Umbilical vein endothelial cells (HUVEC) treated with VEGF-A or EGF for up to 6 hours. VEGF-A is a major trigger of vasculogenesis and angiogenesis. | no change | 1.00 | - | NO CHANGE | 1.00 | - |
| [GDS855](http://www.ncbi.nlm.nih.gov/sites/GDSbrowser?acc=GDS855) | CB-CD34 CD34+ hematopoietic stem cells after treatment with 200 pM TGFbeta for 2 and 4 hours | no change | 1.00 | - | NO CHANGE | 1.00 | - |
| [GDS854](http://www.ncbi.nlm.nih.gov/sites/GDSbrowser?acc=GDS854) | M091 acute myelogenous leukemia cells after treatment with 200 pM transforming growth factor beta (TGFbeta) for 2 and 4 hours. | no change | 1.00 | - | No data | | |
| [GDS2146](http://www.ncbi.nlm.nih.gov/sites/GDSbrowser?acc=GDS2146) | Astrocytes 4 or 12 hours after epidermal growth factor (EGF) treatment to activate the EGF receptor (EGFR). | no change | 1.00 | - | NO CHANGE | 1.00 | - |
| [GDS1549](http://www.ncbi.nlm.nih.gov/sites/GDSbrowser?acc=GDS1549) | Estrogen receptor positive breast cancer cell lines treated with estradiol for 24 hours. MCF-7, T47-D, and BT-474 breast cancer cell lines examined. | no change | 1.00 | - | Increase in MCF-7 after estradiol treatment | 1.24 | 0.013747 |
| [GDS2932](http://www.ncbi.nlm.nih.gov/sites/GDSbrowser?acc=GDS2932) | Normal lung epithelial HPL1D cells and lung adenocarcinoma A549 cells treated with TGF-beta for up to 12 hours. TGF-beta inhibits growth and promotes apoptosis in normal epithelial cells but acts as a pro-tumor cytokine by promoting tumour angiogenesis, immune-escape, and metastasis. | no change | 1.00 | - | NO CHANGE | 1.00 | - |
| [GDS2622](http://www.ncbi.nlm.nih.gov/sites/GDSbrowser?acc=GDS2622) | Mammary epithelial MCF10A cells following treatment with epidermal growth factor for various time points up to 480 minutes. | no change | 1.00 | - | NO CHANGE | 1.00 | - |
| [GDS1955](http://www.ncbi.nlm.nih.gov/sites/GDSbrowser?acc=GDS1955) | Mammary glands from transgenic animals treated with dimerizing agent AP20187 to activate inducible fibroblast growth factor receptor 1 (iFGFR1). iFGFR1 dimerization induces proliferation in the mammary gland. | no change | 1.00 | - | No data | | |
| [GDS2626](http://www.ncbi.nlm.nih.gov/sites/GDSbrowser?acc=GDS2626) | MCF-7 breast cancer cells treated with various doses of epidermal growth factor (EGF) or heregulin (HRG) for up to 90 minutes. | no change | 1.00 | - | NO CHANGE | 1.00 | - |
| [GDS1109](http://www.ncbi.nlm.nih.gov/sites/GDSbrowser?acc=GDS1109) | Expression profiling of blastocysts following inhibition of JNK, p38 MAPK, or ERK signaling. JNK, p38 MAPK, and ERK signaling inhibited by SP600125, SB203580, and U0126 respectively. Results provide insight into the role of MAPK pathways in preimplantation development. | variable | - | - | variable | - | - |
