# Supplementary material for: Expression of FACT in mammalian tissues suggests its role in maintaining of undifferentiated state of cells
Source: Oncotarget. 2011 Oct 13;2(10):783–96. doi: 10.18632/oncotarget.340 (PMC3248156; doi:10.18632/oncotarget.340)
Supplement: Supplementary file 7 [file oncotarget-02-783-s007.docx]

| **Table S4. Summary of studies in which levels of FACT subunits were measured in cells or organs expressing different levels of oncogenes.** | | | | | | | | |
| --- | --- | --- | --- | --- | --- | --- | --- | --- |
|  |  |  |  |  |  |  |  |  |
| **GEO Dataset Study** | **Description of experiment** | **SSRP1** | | | **SPT16 (=Supt16h)** | | | **Oncogene involved** |
|  |  | **description of change** | **fold change** | **p-value** | **description of change** | **fold change** | **p-value** |  |
| [GDS2406](http://www.ncbi.nlm.nih.gov/sites/GDSbrowser?acc=GDS2406) | Lungs of embryonic day 18.5 transgenic embryos overexpressing N-myc in the lung epithelia. | higher in N-myc cells | 2.1 | 0.006 | higher in N-myc cells | 1.433333 | 0.0054 | N-myc |
| [GDS3008](http://www.ncbi.nlm.nih.gov/sites/GDSbrowser?acc=GDS3008) | Medulloblastoma tumors formed in transgenics by expressing an activated form of Smoothened (Smo). | higher in tumors | 3 | 0.04 | higher in N-myc cells | 1.8 | 0.003 | Smoothed |
| [GDS1078](http://www.ncbi.nlm.nih.gov/sites/GDSbrowser?acc=GDS1078) | SKMEL-2 melanoma cells overexpressing the transcription factor E2F-1. E2F-1 overexpression induces apoptosis in many tumor cells | no change | 1 | - | No data | | | E2F1 |
| [GDS3321](http://www.ncbi.nlm.nih.gov/sites/GDSbrowser?acc=GDS3321) | Cancerous lungs of transgenics overexpressing the c-Myc proto-oncogene in their alveolar epithelia | no change | 1 | - | insignificantly lower in c-myc cells | 1.333333 | 0.2 | c-Myc |
| [GDS1211](http://www.ncbi.nlm.nih.gov/sites/GDSbrowser?acc=GDS1211) | Embryonic fibroblasts overexpressing mTert, the catalytic subunit of telomerase | no change | 1 | - | variable | - | - | mTERT |
| [GDS1272](http://www.ncbi.nlm.nih.gov/sites/GDSbrowser?acc=GDS1272) | Thoracic mammary gland from luteinizing hormone (LH) overexpressing transgenic virgins at 16 weeks of age. | no change | 1 | - | No data | | | LH |
| [GDS1306](http://www.ncbi.nlm.nih.gov/sites/GDSbrowser?acc=GDS1306) | Hearts after induction of activated transgene mutants of Ras, MKK3, or MKK7, which are specific upstream activators of the MAP kinases ERK, p38, and JNK respectively. Hearts examined at various time points up to 4 weeks following each transgene induction using tamoxifen. | higher in Ha-Ras mutant hearts at 4 to 7 days | 1 | - | highest in Ha-Ras mutant hearts | 1.666667 | 0.0065 | Ha-Ras |
| [GDS1637](http://www.ncbi.nlm.nih.gov/sites/GDSbrowser?acc=GDS1637) | Transformed cell lines that either exhibit oncogene-induced senescence (OIS) triggered by MEK activation or bypass OIS. Senescent cells exist in premalignant tumors but not in malignant ones. Results provide insight into the molecular basis of OIS. | control<senescence<senescence bypassed | 2.9 | 4E-05 | no change | - | - | MEK |
| [GDS1630](http://www.ncbi.nlm.nih.gov/sites/GDSbrowser?acc=GDS1630) | Analysis of EA.hy926, an immortalized umbilical vein endothelial cell (HUVEC) line, and its primary cell counterpart. | higher in immortalized cells | 5.1 | 0.0001 | higher in immortalized cells | 1.320755 | 0.0023 | fusion with tumor A549 cells |
| [GDS3319](http://www.ncbi.nlm.nih.gov/sites/GDSbrowser?acc=GDS3319) | Analysis of transgenic (Tg) thyroids expressing RET/PTC3 fusion gene or E7 human papillomavirus 16 oncoprotein. | higher in Tg | 1.2 | ###### | higher in Tg | 1.24 | 0.0005 | E7 fusion proteins |
| [GDS3455](http://www.ncbi.nlm.nih.gov/sites/GDSbrowser?acc=GDS3455) | Analysis of MLP-29 liver cells bearing the heterozygous Kras G12D mutation. | slighly higher in K-ras mutants | 1.0294 | 0.003 | slighly higher in K-ras mutants | variable | - | K-ras |
| [GDS1222](http://www.ncbi.nlm.nih.gov/sites/GDSbrowser?acc=GDS1222) | Analysis of cancer progression by profiling of preneoplastic mammary glands and tumors of MMTV-neu animals. | variable | - | - | No data | | | Her2 |
| [GDS3550](http://www.ncbi.nlm.nih.gov/sites/GDSbrowser?acc=GDS3550) | Analysis of kidney epithelial RK3E cells transformed by the oncogenic transcription factor GLI1. | higher in Gli1 cells | 1.375 | 0.0002 | higher in Gli1 cells | 1.290323 | 0.06 | Gli |
| [GDS2514](http://www.ncbi.nlm.nih.gov/sites/GDSbrowser?acc=GDS2514) | Analysis of mammary gland segments from WAP–SVT/t transgenic animals. These animals selectively synthesize the SV40 T/t antigen in mammary gland epithelial (ME) cells and develop breast cancer after the first lactation. | higher in transgenic animals | 2.35 | 0.0005 | higher in transgenic animals | 1.8 | 0.03 | SV40 |
